# Activity of Compounds from Temperate Propolis against *Trypanosoma brucei* and *Leishmania mexicana*

**DOI:** 10.3390/molecules26133912

**Published:** 2021-06-26

**Authors:** Adullah Alotaibi, Godwin U. Ebiloma, Roderick Williams, Ibrahim A. Alfayez, Manal J. Natto, Sameah Alenezi, Weam Siheri, Malik AlQarni, John O. Igoli, James Fearnley, Harry P. De Koning, David G. Watson

**Affiliations:** 1 Strathclyde Institute of Pharmacy and Biomedical Science, University of Strathclyde, 161 Cathedral Street, Glasgow G4 0RE, UK; abdullah.alotaibi@strath.ac.uk (A.A.); sameahq8@gmail.com (S.A.); j.o.igoli@uam.edu.ng (J.O.I.); 2 Institute of Infection, Immunity and Inflammation, College of Medical, Veterinary and Life Sciences, University of Glasgow, Glasgow G12 8TA, UK; g.ebiloma@tees.ac.uk (G.U.E.); ibrahim-fa@hotmail.com (I.A.A.); manal.natto@glasgow.ac.uk (M.J.N.); 3 School of Health and Life Sciences, Teesside University, Middlesbrough TS1 3BX, UK; 4 IBEHR, School of Health and Life Science, University of the West of Scotland, High Street, Paisley PA1 2BE, UK; roderick.williams@uws.ac.uk; 5 Qassim Health Cluster, Ministry of Health, Buraydah 52367, Saudi Arabia; 6 Department of Pharmaceutical Chemistry, College of Clinical Pharmacy, Imam Abdulrahman Bin Faisal University, Dammam 31441, Saudi Arabia; malquarni876@gmail.com; 7 Department of Pharmacognosy and Natural Products, Faculty of Pharmacy, University of Tripoli, Tripoli 50676, Libya; weamsiheri@gmail.com; 8 Department of Chemistry, University of Agriculture, Makurdi PMB 2373, Nigeria; 9 BeeVital, Whitby, North Yorkshire YO22 5JR, UK; james.fearnley@beevitalpropolis.com

**Keywords:** temperate propolis, flavonoids, isolated compounds, *Trypanosoma brucei*, *Leishmania mexicana*

## Abstract

Ethanolic extracts of samples of temperate zone propolis, four from the UK and one from Poland, were tested against three *Trypanosoma brucei* strains and displayed EC_50_ values < 20 µg/mL. The extracts were fractionated, from which 12 compounds and one two-component mixture were isolated, and characterized by NMR and high-resolution mass spectrometry, as 3-acetoxypinobanksin, tectochrysin, kaempferol, pinocembrin, 4′-methoxykaempferol, galangin, chrysin, apigenin, pinostrobin, cinnamic acid, coumaric acid, cinnamyl ester/coumaric acid benzyl ester (mixture), 4′,7-dimethoxykaempferol, and naringenin 4′,7-dimethyl ether. The isolated compounds were tested against drug-sensitive and drug-resistant strains of *T. brucei* and *Leishmania mexicana*, with the highest activities ≤ 15 µM. The most active compounds against *T. brucei* were naringenin 4′,7 dimethyl ether and 4′methoxy kaempferol with activity of 15–20 µM against the three *T. brucei* strains. The most active compounds against *L. mexicana* were 4′,7-dimethoxykaempferol and the coumaric acid ester mixture, with EC_50_ values of 12.9 ± 3.7 µM and 13.1 ± 1.0 µM. No loss of activity was found with the diamidine- and arsenical-resistant or phenanthridine-resistant *T. brucei* strains, or the miltefosine-resistant *L. mexicana* strain; no clear structure activity relationship was observed for the isolated compounds. Temperate propolis yields multiple compounds with anti-kinetoplastid activity.

## 1. Introduction

Propolis, generally referred to as ‘bee glue’, is the generic name given to the natural, resin-like substance that is produced by bees. It is made from a mixture of bee saliva, beeswax, and plant exudate/secretions gathered from tree buds, sap flows, or other botanical sources collected by the bees during visits to various types of vegetation [1,2,3]. Propolis is a very versatile, multifunctional substance with several applications in the hive such as the sealing of holes and cracks to the general reconstruction of the beehive. It is used to coat the inner surface of the beehive, helping to regulate the hive’s internal temperature keeping it around an average of 35 °C [4]. It also guards against weather damage and serves to prevent against the invasion of the colony by predators. The propolis helps to provide an anti-microbial environment within the hive protecting larvae, honey stores etc., from bacterial and fungal infections [5,6] and it also has activity against protozoan parasites [2]. The chemical composition of propolis samples determines their biological properties and is dependent on factors such as the season, geographic origin of the samples and plant resources in the proximity of the hive. In Europe, the resinous exudate produced by the buds of poplar trees is the primary component of bee propolis. European propolis is rich in flavones and flavanones, phenolic acids, and their esters when compared with propolis samples from tropical regions, which usually contain mainly prenylated flavonoids and terpenes [1].

Human African trypanosomiasis (HAT, sleeping sickness) and animal African trypanosomiasis (AAT, nagana) are diseases caused by infection with parasitic protozoa of the genus *Trypanosoma*. The infection is mostly spread as a result of bites from the tsetse fly in sub-Saharan Africa but some *Trypanosoma* species have adapted to transmission by other biting insects (*T. evansi*, *T. vivax*) or sexual transmission (*T. equiperdum*); as a result, those species have spread to southern and central Asia, and to South America [7].

For 40 years, there was no significant development in the treatment of these diseases, particularly the veterinary conditions, which are currently managed by only by a limited number of anti-microbials to which over time the parasites have become resistant [8,9,10]. There is mounting evidence that bees become infected with trypanosomatid parasites although the impact of these infections on bee health is still unclear [11,12,13,14,15,16]. Trypanosomatids are probably transmitted in the hive via faeces of infected bees [17] and coating the hive surface with propolis that has anti-trypanosomal activity could therefore act to prevent transmission. In previous work it has been observed that propolis has activity against trypanosomatids [18,19,20,21], and this has recently been comprehensively reviewed [22]. In this paper, we follow on from our earlier paper, which profiled the anti-trypanosomatid activity of temperate propolis samples from various European countries [23], by isolating and testing some of the compounds present in some UK and Polish propolis samples against trypanosomes and *Leishmania*.

## 2. Results

### 2.1. Extracts

The results for the extraction of the propolis samples are summarized in Table 1.

### 2.2. Testing of Crude Extracts against Trypansomes

The ethanolic extracts of the UK propolis samples were tested against a standard drug sensitive laboratory strain of *T. b. brucei* (s427) and a derived multi-drug resistant *T. b. brucei* clone (B48), using pentamidine as a control drug, and the EC_50_ values were calculated. The results show that the four-samples exhibited variable significant activity against *T. brucei* except for S225 which showed moderate activity against *T. brucei* with EC_50_ > 10 µg/mL. The crude samples S224, D6 and D7, had variable but significant activity against *T. brucei* with EC_50_ values of 5.28, 4.49, and 5.97 µg/mL, respectively as shown in Table 2.

### 2.3. Characterisation of Compounds Isolated from Propolis

#### 2.3.1. Characterisation of Pinobanksin-3-*O*-Acetate

The compound was obtained as a yellowish solid from a Grace Reveleris (Deerfiled, IL, USA) MPLC fraction. The compound appeared as a purple spot on TLC after spraying with anisaldehyde sulfuric acid reagent and as a dark spot under UV of 254 nm and a yellow spot under UV of 365 nm. The exact mass obtained from LC-HRMS gave an [M − H]^−^ ion at *m/z* = 313. 0728 (Figure S2, Supplementary Materials). Hydrogen and carbon atoms in the compound were assigned as in Table S1 (Supplementary Materials), the ^1^H and ^13^C spectra are shown in Figures S3 and S4 (Supplementary Materials). The identity was confirmed by comparison of its chemical shift data with literature reports [27]. Pinobanksin-3-*O*-acetate (Figure S1, Supplementary Materials) was previously isolated from Jordanian propolis and tested in vitro for antibacterial activity; it was found to exhibit activity against methicillin resistant *Staphylococcus aureus* (MRSA) and *E. coli* [27].

#### 2.3.2. Characterization of Tectochrysin

The compound was obtained as a white amorphous solid from a Grace Reveleris MPLC fraction. The positive mode LC-HRMS spectrum of the compound; Figure S6 (Supplementary Materials) gave a molecular ion [M + H]^+^ at *m/z* 269.0790 suggesting a molecular formula of C_16_H_12_O_4_ (Calc for (C_16_H_13_O_4_), 269.0814). Hydrogen and carbon atoms in the compound were assigned as in Table S2 (Supplementary Materials), the ^1^H and ^13^C spectra are shown in Figures S7 and S8 (Supplementary Materials). The structure of the compound was confirmed as tectochrysin (Figure S5, Supplementary Materials) and the chemical shifts for the protons and carbon atoms are given in Table S2 alongside literature reports [28,29].

#### 2.3.3. Characterization of Faempferol

The compound was obtained as a yellowish solid from a Grace Reveleris MPLC fraction followed by further purification on Sephadex LH20. The negative mode HRESI-MS spectrum of the compound gave a molecular ion [M − H]^−^ at *m/z* 285.0403 (Figure S10, Supplementary Materials), (Calcd 285.0399 for C_15_H_9_O_6_) suggesting a molecular formula of C_15_H_10_O_6_. Hydrogen and carbon atoms in the compound were assigned as in Table S3 (Supplementary Materials), the ^1^H and ^13^C spectra are shown in Figures S11 and S12, Supplementary Materials. The compound was identified as kaempferol (Figure S9, Supplementary Materials) and confirmed by literature reports [30].

#### 2.3.4. Characterization of Pinocembrin

The compound was obtained as yellow needle-shaped crystals from a Grace Reveleris MPLC fraction followed by further purification on Sephadex LH20. The negative mode HRESI-MS spectrum of the compound gave a molecular ion [M − H]^−^ at *m/z* 255.0665 (C_15_H_11_O_4_) (Calc for 255.0657), suggesting a molecular formula of C_15_H_12_O_4_ (Figure S14). Hydrogen and carbon atoms in the compound were assigned as in Table S4, the ^1^H and ^13^C spectra are shown in Figures S15 and S16. Further examination of its spectra identified the compound as pinocembrin (Figure S13) and this was confirmed by literature reports [30]. Pinocembrin is considered the most abundant compound in temperate propolis. Its biological activities have been evaluated, and include antioxidant, anti-inflammatory, and antimicrobial properties among its many pharmacological activities [31]. It has been isolated from Mexican brown and red propolis [32,33].

#### 2.3.5. Characterization of 4′-Methoxykaempferol

The compound was obtained as a light-yellow solid from column chromatography followed by further purification on Sephadex^®^ LH20 (Sigma-Aldrich, Gillingham, Dorset, UK). The positive mode HRESI-MS spectrum of the compound gave a molecular ion [M + H] ^+^ at *m/z* 301.0701 (Calc for (C_16_H_13_O_6_), 301. 0712) suggesting a molecular formula of C_16_H_12_O_6_ (Figure S18). Hydrogen and carbon atoms in the compound were assigned as in Table S5, the ^1^H and ^13^C spectra are shown in Figures S19 and S20. The compound was confirmed as 4′-methoxykaempferol (Figure S17) by comparison with the literature reports [34].

#### 2.3.6. Characterization of Galangin

The compound was obtained as yellow needles after column chromatography followed by purification on Sephadex LH20. The negative mode HRESI-MS spectrum of the compound gave a molecular ion [M − H]^−^ at *m/z* 269.0428 (Calc for (C_15_H_9_O_5_), 269.0450) suggesting a molecular formula C_15_H_10_O_5_ (Figure S22). Hydrogen and carbon atoms in the compound were assigned as in Table S6, the ^1^H and ^13^C spectra are shown in Figures S23 and S24. The compound was identified as galangin (Figure S21) and confirmed by literature reports [30]. The chemical shifts for the protons and carbon atoms are given in Table S6. Galangin was previously isolated from propolis from the north of Argentina [35].

#### 2.3.7. Characterization of Chrysin

The compound was obtained as a pale-yellow solid from column chromatography followed by purification on Sephadex LH20. The positive mode HRESI-MS spectrum of the compound gave a molecular ion [M + H]^+^ at *m/z* 255.0632 (Calc for (C_15_H_11_O_4_), 255.0657) suggesting a molecular formula C_15_H_10_O_4_ (Figure S26). Hydrogen and carbon atoms in the compound were assigned as in Table S7, the ^1^H and ^13^C spectra are shown in Figures S27 and S28. The compound was identified as chrysin (Figure S25) and confirmed by literature reports [30].

#### 2.3.8. Characterization of Apigenin

The compound was obtained as a pale-yellow solid after column chromatography followed by purification on Sephadex LH20. The negative mode HRESI-MS spectrum of the compound gave a molecular ion [M − H]^−^ at *m/z* 269.0454 (Calc for (C_15_H_9_O_5_), 269.0450), suggesting a molecular formula C_15_H_10_O_5_ (Figure S30). Hydrogen and carbon atoms in the compound were assigned as in Table S8, the ^1^H and ^13^C spectra are shown in Figures S31 and S32. The compound was identified as apigenin (Figure S29) and confirmed by literature reports [30]. Apigenin was previously isolated from a crude chloroform extract of *Moquinia kingie* and tested against *Trypanosoma cruzi*; it was found to have activity against trypomastigotes in vitro [36].

#### 2.3.9. Characterization of Pinostrobin

The compound was obtained as a dark yellow solid after column chromatography followed by further purification on Sephadex LH20. The positive mode HRESI-MS spectrum of the compound gave a molecular ion [M + H]^+^ at *m/z* 271.0939 (Calc for (C_16_H_15_O_4_), 271. 0970), suggesting a molecular formula of C_16_H_14_O_4_ (Figure S34). Hydrogen and carbon atoms in the compound were assigned as in Table S9, the ^1^H and ^13^C spectra are shown in Figures S35 and S36. The compound was identified as pinostrobin (Figure S33) and confirmed by literature reports [30].

#### 2.3.10. Characterization Cinnamic Acid

The compound was obtained as a dark yellow solid from column chromatography followed by further purification on Sephadex LH20. The positive mode HRESI-MS spectrum of the compound gave a molecular ion [M + H]^+^ at *m/z* 149.0587 (Calc for (C_9_H_9_O_2_), 149.0602) suggesting a molecular formula of C_9_H_8_O_2_ (Figure S38). Hydrogen and carbon atoms in the compound were assigned as in Table S10, the ^1^H and ^13^C spectra are shown in Figures S39 and S40. The compound was identified as cinnamic acid (Figure S37) and confirmed by literature reports [30].

#### 2.3.11. Characterization of Coumaric Acid Cinnamyl Ester as Part of a Mixture

The compound was obtained as a light-yellow powder after column chromatography followed by further purification on Sephadex LH20. The negative mode HRESI-MS spectrum gave a molecular ion [M − H]^−^ at m/z 279.0998 (Calc for (C_18_H_15_O_3_), 279.102120), suggesting a molecular formula of C_18_H_16_O_3_ (Figure S42). Hydrogen and carbon atoms in the compound were assigned as in Table S11, the ^1^H and ^13^C spectra are shown in Figures S43 and S44. The component was identified as coumaric acid cinnamyl ester (Figure S41) and confirmed by literature reports [37].

#### 2.3.12. Characterization of Coumaric Acid Benzyl Ester as Part of Mixture

The compound was obtained as a light-yellow powder after column chromatography followed by further purification on Sephadex LH20. The positive mode HRESI-MS spectrum of the compound gave a molecular ion [M + H]^+^ at *m/z* 255.0996 (Calc for (C_16_H_15_O_3_), 255.1021), suggesting a molecular formula of C_16_H_14_O_3_ (Figure S46). Hydrogen and carbon atoms in the compound were assigned as in Table S12, the ^1^H and ^13^C spectra are shown in Figures S43 and S44. The component was identified as coumaric acid benzyl ester (Figure S45) and confirmed by literature reports [37].

#### 2.3.13. Characterization of 4′,7-Dimethoxykaempferol

The compound was obtained as a yellow powder after column chromatography and Sephadex column chromatography. The positive mode HRESI-MS spectrum of the compound gave a molecular ion [M + H] ^+^ at *m/z* 315.0838 (Calc for (C_17_H_15_O_6_), 315.0869), suggesting a molecular formula of C_17_H_14_O_6_ (Figure S48). Hydrogen and carbon atoms in the compound were assigned as in Table S13, the ^1^H and ^13^C spectra are shown in Figures S49 and S50. The compound was identified as 4′,7-dimethoxykaempferol (Figure S47) and confirmed by literature reports [38,39].

#### 2.3.14. Characterization of Naringenin 4′,7-Dimethyl Ether

The compound was obtained as a pale-yellow solid after column chromatography and Sephadex column chromatography. The positive mode HRESI-MS spectrum of the compound gave a molecular ion [M + H] ^+^ at *m/z* 301.10443 (Calc for (C_17_H_17_O_5_), 301.1076), suggesting a molecular formula of C_17_H_16_O_5_ (Figure S52). Hydrogen and carbon atoms in the compound were assigned as in Table S14, the ^1^H and ^13^C spectra are shown in Figures S53 and S54. The compound was identified as naringenin 4′,7-dimethyl ether (Figure S51) and confirmed by literature reports [39].

### 2.4. Biological Activity for the Compounds Isolated from Temperate Propolis Samples against T. brucei and L. mexicana

The pure compounds isolated from these samples were tested against the standard drug-sensitive strain *T. brucei* (s427WT) [40], diamidine and melaminophenyl-arsenical cross-resistant B48 [26], isometamidium, and pentamidine-resistant strain *T. b. brucei* ISMR1 [41], with pentamidine as a control drug. When drug sensitivities were calculated the results showed that most of the pure compounds isolated exhibited moderate activity against all *T. brucei* isolates, with EC_50_ values for 5 compounds ≤ 25 µM (Table 3).

Differences between the WT and the two multi-drug resistant strains were small, not exceeding ~1.5-fold, compared to 196-fold and 21.8-fold resistance to the reference drug pentamidine for B48 and ISMR1, respectively.

For *L. mexicana* WT and the miltefosine-APC12 resistant strain *L. mexicana* C12Rx [23], the results from drug sensitivities showed that most of the pure compounds isolated exhibited variable but significant activity related to the structure of the compounds (Table 4). Miltefosine was used as the control drug.

For antitrypanosomal activity against TbS427WT, the flavones appear to be more active than the flavans. The presence of a hydroxy substituent at C-3 and a methoxy at C-4′ seem to be important for activity but more important is the C-4′-OCH_3_. Hence 4-methoxykaempferol (15.2 µM) is more active than naringenin-4′,7-dimethyl ether (17.5 µM) and kaempferol (24.0 µM). For activity against *L. mexicana* WT, the order of activity seems to be altered, and in general there was quite poor correlation between the two sets of EC_50_ values (linear regression, r = 0.237 (*n* = 12); slope not significant non-zero by F-test (*p* = 0.48)). However, pinocembrin, pinostrobin, galangin, apigenin, cinnamic acid, and naringenin 4′,7-dimethyl ether all displayed similar EC_50_s against both of the kinetoplastid parasites (within two-fold).

Kaempferol 4′,7-dimethyl ether (12.9 µM) and coumaric acid cinnamyl or benzyl ester (13.1 µM) were the most active agents against *Leishmania*, followed by chrysin (17.6 µM) and naringenin 4′7-dimethyl ether (17.8 µM). The next active compounds, galangin and apigenin, are flavonoids without the C-3 hydroxy group. The unsubstituted phenyl ring in chrysin and galangin still confers more activity than the flavanones, flavanonols or 3-hydroxyflavanones, with naringenin-4′,7-dimethyl ether being the only flavanone that showed any significant activity. The coumaric acid ester was only significantly active in the antileishmanial but not in the antitrypanosomal assays. Cinnamic acid itself did not show significant antitrypanosomal (62.2 µM) or antileishmanial (111.0 µM) activity compared to the ester (45.8 µM and 13.1 µM, respectively).

## 3. Discussion

In this work, 12 compounds and a two-component mixture were isolated from four propolis samples from the UK and two compounds were isolated from Polish propolis. Most of these compounds were identified in a previous study in propolis from two different geographical locations of Northern Italy, which identified a number of flavonoids including pinocembrin, chrysin, galangin, pinobanskin-3-*O*-acetate and pinostrobin, as well as several phenolic acids, pinobanksin, kaempheride, and apigenin [42]. Another study determined that the same classes of compounds were abundant in a sample of propolis from New Zealand [43]. A study on 10 propolis samples from Bulgaria, Italy, and Switzerland, analysed by GC-MS, found that most of the compounds were typical of poplar buds [44]. This data was in agreement with an earlier study for temperate poplar propolis from Turkey [45], suggesting that this might be the source of propolis in temperate zones.

Focusing on the isolated compounds that have antiprotozoal activity against *T. brucei* WT and two different drug-adapted strains, allowed testing for cross-resistance against some of the most important drugs against HAT (pentamidine, melarsoprol (B48)) and veterinary trypanosomiasis (diminazene, cymelarsan (B48), isometamidium, and homidium (ISMR1)) [26,41]. All the compounds exhibited EC_50_ values of < 100 µM (apart from tectochrysin) against the standard drug-sensitive strain of *T. brucei*, s427, with highest activity observed for 4′-methoxykaempferol (15.2 ± 0.4 µM). Comparison of the EC_50_ values with those of the drug resistant strains showed only minor variation with less than 1.5-fold lower activity against B48 or ISMR1, confirming our previous conclusion that there is little or no prospect of cross-resistance between current anti-trypanosomal agents and propolis-derived natural compounds [18,19,20,21]. In *Trypanosoma brucei*, most drug resistance is linked to changes in drug uptake [46,47], involving the TbAT1 aminopurine transporter (diminazene [25,48]), amino acid transporter TbAAT6 (for eflornithine [49]) and aquaglyceroporin TbAQP2 (pentamidine, melaminophenyl arsenicals [47,50]), but the compounds isolated from propolis are structurally unrelated to the substrates of those transporters [48,50] and thus not expected to be substrates, explaining the lack of cross-resistance.

The most active compounds against *T. brucei* were 4′-methoxykaempferol and naringenin 4′,7-dimethyl ether. No activity was observed for tectochrysin. Galangin and apigenin are quite active with similar EC_50_ values and are isomers, having the same number of hydroxyl groups. Chrysin and pinocembrin have the same number of hydroxyl groups and relatively low activity. It is hard to see clear structure activity relationships and the activities may depend on hydrophilic-lipophilic balance, although naringenin 4′,7-dimethyl ether with its high activity does not fit this theory since with two methoxy groups and a partially unsaturated C ring it is relatively lipophilic in comparison to 4′ methoxykaempferol. The activities of just over half the compounds were similar against *L. mexicana* and *T. brucei*, but kaempferol and 4′-methoxykaempferol were substantially more active against *T. brucei* whereas chrysin

Kaempferol 4′ 7-dimethyl ether and the coumaric acid esters were more clearly active against *L. mexicana*. The lack of consistent correlation and the clear emergence of a structure-activity relationship would be consistent with (some of) the compounds having different mechanisms of action on the parasites.

Although many of these compounds were previously isolated from propolis samples from different areas such as Jordan [27] and Poland [51], this is the first study that has isolated and characterised constituents of ethanolic extracts of propolis samples from the UK and investigated their anti-protozoal activity. Propolis samples from Bulgaria were previously evaluated for their activity against *Trypanosoma cruzi* and the acetone and ethanol extracts of two samples showed strong inhibitory activity against that trypanosomatid [52]. The activities of the coumaric acid esters (cinnamyl and benzyl) against *L. mexicana* should be further investigated with pure compound and the SAR expanded upon. This class of compound, commonly present in the diet and generally accepted as having low toxicity, should be further investigated for anti-protozoal activity.

## 4. Materials and Methods

### 4.1. Chemicals and Laboratory Materials

HPLC grade solvents including hexane, ethyl acetate, methanol, acetonitrile and absolute ethanol were obtained from Fisher Scientific (Loughborough, UK). AnalaR grade formic acid (98%) was from BDH-Merck (Dorset, UK) and HPLC grade water was produced in-house using a Milli Q system water purifier from Millipore, UK.

Other materials were obtained as follows: 4ml glass vials 45 × 14.75 mm (Kinesis Ltd., St. Neots, Cambridgeshire, UK), ACE C18 column (3 × 150 mm, 3 µm) (Hichrom, UK), the syringes and Acrodisc filters, ultrasonic Bath (Scientific Laboratory Supplies, Ltd., Nottingham, UK), NMR tubes (5 mm 300 MHz, 187 mm L, from Norell^TM^ (obtained through Fisher Scientific (Loughborough, UK)). Glass columns for column chromatography were from Rotaflo (Whaley Bridge, Derbyshire, UK). Empty dry-loader cartridges for samples packing to be loaded onto the Grace Reveleris system (Grace Reveleris iES Chromatography System from Alltech, Carnforth, Lancs, UK), C18 (12 g) cartridges and silica cartridges (24 g) were from VWR (Leicesteshire, UK). The NMR analyses were run in deuterated solvents including CDCl_3_, DMSO-*d*6, and Acetone-*d*6, purchased from Sigma-Aldrich (Dorset, UK).

The UK propolis samples were provided by BeeVital (Whitby, UK) while a sample of Polish propolis were purchased from Amazon UK.

### 4.2. Chromatography

Silica gel of mesh size 200–425 µm, for column chromatography, Davisil grade 633 amorphous precipitated silica of pore size 60 A, and Celite filter agent for sample dry loading onto MPLC were purchased from Sigma-Aldrich (Dorset, UK). Davisil grade 636 column grade silica gel pore size 60A, mesh size 35–60 and TLC silica gel 60 F254 pre-coated aluminium sheets were obtained from Merck (Darmstadt, Germany) and was used for column chromatography and thin layer chromatography, respectively.

### 4.3. Extraction of Propolis Samples

The propolis samples were extracted with 100 mL ethanol by sonication at 40 °C for 1 h each. The amounts extracted are shown in Table 1. They were then spun on a centrifuge at 2500 rpm for 15 min. Each sample was then filtered, and the filtrate collected. The filtered solids were re-extracted twice with ethanol (100 mL). The combined filtrates (EEP) were then dried by rotary evaporation. The weight of each extract was determined before storage at −20 °C.

### 4.4. General Profiling of Crude Samples of Propolis

The dried ethanol extracts of the propolis samples were dissolved in methanol (HPLC grade) to obtain a concentration of 1 mg/mL, the samples were filtered using a syringe filter (Acrodisc 0.45 μm) and 10 µL samples were injected into an UltiMate-3000 HPLC system connected to an Orbitrap Exactive MS (Thermo Fisher Scientific, Hemel Hempstead, UK). The mobile phase was composed of 0.1% formic acid in acetonitrile as solvent A and 0.1% formic acid in water as solvent B and set at a flow rate of 300 µL/min (Table 5). The high-resolution mass spectra were obtained using with a needle voltage of 4.5 kV in positive and −4kV in negative mode. The liquid chromatography was performed on an ACE-C18 column (150 × 3 mm, 3 µm) from HiChrom UK. The MS detection range was from m/z 100 to 1500 and the scanning was performed under ESI polarity switching mode to permit acquisition of positive and negative ion data in a single experiment. Samples containing the purified compounds were dissolved (100 μg/mL) in methanol.

### 4.5. Fractionation of Extracts

Four samples from the UK (S224) from the Midlands, UK, S225 from Essex, D6 from Northhamptonshire, D7 from Essex, UK and a sample from Poland (P) were extracted and subjected to preparative scale fractionation.

In this study, two methods (A and B) were used for the fractionation of the propolis samples.

Method A, the ethanol extract was subjected to column chromatography (CC) (General method 1).

Fractions were combined based on their LCMS profiling results. Fractions with over 100 mg of material were then subjected to fractionation and purification using Medium Pressure Liquid Chromatography MPLC (General Method 4). The fractions from MPLC, based on their LCMS profiling, were combined and subjected to further purification using gel filtration chromatography (General method 3).

Method B involved partitioning between dichloromethane and hexane. The dried ethanol extract was dissolved in 200 mL of dichloromethane and filtered. Then 500 mL of hexane was added to the dichloromethane soluble portion. This caused immediate precipitation and the mixture was filtered to obtain a clear filtrate and residue. The filtrate was allowed to dry in a fume hood. The residue and dried filtrate were subjected to column chromatography and elution was started with hexane:ethyl acetate (80:20) (General method 1) or vacuum liquid chromatography (General method 2) and gel filtration chromatography (General method 3).

#### 4.5.1. General Method 1: Column Chromatography

A sample (2 g) of the ethanol extract of the propolis was dissolved in ethyl acetate. The solidified wax and the residue were removed by filtration, and the filtrate was evaporated to dryness and mixed with 5 g of coarse silica in a beaker, and the solvent was removed under a fume hood. Then silica gel 60 with a mesh size of 200–425 μm (50 g) was mixed with 200 mL of hexane and used to pack a glass column (55 × 3 cm). The sample mixed with silica was loaded onto the top of the column and elution was carried out as follows (collecting fractions in 50 mL flasks): 200 mL of hexane, F1; then 200 mL of hexane/ethyl acetate (80:20) F2; then 200 mL of hexane/ethyl acetate (60:40) F3; 200 mL of hexane/ethyl acetate (40:60) F4; 200 mL of hexane/ethyl acetate (20:80) F5; 200 mL of ethyl acetate (100%) F6; and finally, 200 mL of ethyl acetate/methanol (70:30) F7. This produced 28 fractions, which were dried and weighed. They were pooled according to their LC-MS profiling results and based on similarities in their abundance of compounds.

#### 4.5.2. General Method 2: Vacuum Liquid Chromatography (VLC)

Dry silica gel 60H was loaded on a glass Büchner funnel (13 cm (d) × 10 cm (h)) under vacuum. Dried propolis extracts (10 g) were dissolved in 25 mL of methanol and adsorbed on a small amount of silica gel 60. The adsorbed sample was left to dry and thereafter loaded on the top of the column. The column was eluted starting with, *n*-hexane/EtOAc and EtOAc/MeOH mixtures as follows (collecting fractions in 500 mL round bottom flasks): 500 mL of hexane/ethyl acetate (70:30) F1; then 500 mL of hexane/ethyl acetate (50:50) F2; 500 mL of hexane/ethyl acetate (30:70) F3; 500 mL of hexane/ethyl acetate (10:90) F4; 500 mL of ethyl acetate (100%) F5; and finally, 500 mL of ethyl acetate/methanol (70:30) F6.The fractions were evaporated and weighed after drying. They were also examined by LC-MS to determine similar fractions.

#### 4.5.3. General Method 3: Gel Filtration Chromatography (GF)

A slurry of Sephadex LH-20 in methanol was loaded in a glass column (2 cm (id) × 100 cm (l)) and the bed was allowed to settle. The extract to be purified was dissolved in a minimum volume of methanol and applied onto the column. The column was eluted isocratic with methanol after the extract was absorbed into the column bed. Fractions of 3-5 mL were collected, allowed to dry and examined by NMR.

#### 4.5.4. General Method 4: Medium Pressure Liquid Chromatography (MPLC)

MPLC was used to purify crude extracts as well as fractions from column chromatography. Extracts and fractions for purification by MPLC were dissolved in a minimum quantity of ethyl acetate and adsorbed with Celite (1:2 *w*/*w*). The mixture was allowed to dry and subsequently loaded into the specified cartridges. Fractionation was carried out under normal phase conditions using a silica gel column (GraceResolv Silica 24 g/32 mL) or with reversed phase conditions using a C-18 (12 g) cartridge.

#### 4.5.5. Fractionation of Individual Propolis Extracts

##### Fractionation of Sample S224

About 2.0 g of the ethanol extract of S224 was separated by column chromatography (CC) using general method 1. Four fractions (F1–F4), weighing over 120 mg, were further fractionated by medium pressure liquid chromatography (MPLC) on silica gel using method 4 and thereafter profiled by liquid chromatography high resolution mass spectrometry (LC-HRMS) and nuclear magnetic resonance spectroscopy (NMR).

Separation of F1 by MPLC yielded 3-acetoxypinobanksin (13.7 mg), chrysin (3.5 mg) and further separation on Sephadex LH20 yielded 4′-Methoxykaempferol (2.5 mg). Separation of F2 by MPLC yielded galangin (3.0 mg) and further separation using Sephadex LH20 afforded kaempferol (5.0 mg) and 4′-Methoxykaempferol (3.2 mg).

Separation of F3 by MPLC yielded tectochrysin (10.2 mg) and further separation on combined Sephadex LH20 gave pinocembrin (6.5 mg).

##### Fractionation of Sample D7

An amount of the ethanol extract of D7 (2 g) was separated by CC using general method 1, followed by separation on Sephadex LH20. Three compounds were obtained: pinocembrin (4.5 mg), galangin (5.8 mg) and chrysin (5.4 mg).

##### Fractionation of Sample D6

An amount of the ethanol extract of D6 (2 g) was separated by CC using general method 1 followed by separation on a Sephadex LH20 column. Four compounds were obtained: pinocembrin (3.2 mg), galangin (3.1 mg), kaempferol (4.9 mg), and apigenin (6.5 mg).

##### Fractionation of Sample S225

An amount of the ethanol extract of S225 (10 g) was subjected to VLC as described in general method 2. Fraction F1 from the VLC was further subjected to CC as in general method 1. This resulted in the isolation of pinostrobin (14 mg), p-coumaric acid (26.2 mg), and cinnamic acid (24 mg).

##### Fractionation of Sample P

About 600 mg of the ethanol extract of the propolis was subjected to fractionation by Sephadex LH20 according to general method 3. This resulted in the isolation of naringenin 4,7-dimethyl ether (14.0 mg) and kaempferol-4,7-dimethyl ether (16.0 mg).

### 4.6. Thin Layer Chromatography (TLC)

Crude extracts and fractions from column chromatography, gel filtration chromatography and medium pressure liquid chromatography were examined by TLC to compare their profiles and identify similar ones for pooling. TLC were carried out on normal phase pre-coated aluminium backed silica gel plates. The extracts, fractions, and purified compounds were dissolved in an appropriate solvent and applied as a spot on the plate about 2 cm above the base. Plates with samples were developed in suitable solvent systems. After development, the plates were dried, the solvent front was marked and the spots were observed in daylight, UV light, and by spraying with colour-developing reagents.

Spots were observed under short UV (λ 254 nm) where they appeared as dark bands on a green background due to UV quenching and at long UV (λ 366 nm) where fluorescent compounds show as coloured spots. Observed bands were circled or marked with pencil.

Developed TLC plates were also sprayed with p-anisaldehyde-sulfuric acid, vanillin-sulfuric acid. Sprayed plates were heated with a hot-air heater to aid any colour development. The Rf values were calculated and fractions with similar TLC profiles were combined for NMR analysis.

### 4.7. Structure Elucidation

Proton NMR was the main technique used for first-line evaluation and analysis of the extracts and fractions. Those with clean and discernible proton NMR spectra were subjected to ^13^C NMR and where the results indicate a pure compound or identifiable mixtures, 2D NMR spectra were acquired to tease out or elucidate the structures of the compounds or mixtures. The chemical shifts were compared with literature reports to confirm the structures proposed. NMR data were obtained on a JEOL (JNM LA400) spectrophotometer and on a Bruker Avance III (400MHz). For all NMR analysis, TMS was used as an internal standard and deuterated solvents such as CDCl_3_, (CD_3_)_2_CO, and DMSO-*d*6 were used for dissolving the samples. NMR experiments of samples with low quantities were carried out using Shigemi NMR tubes where samples were dissolved in 300 μL of CDCl_3_ or DMSO-*d*6 and placed into a Shigemi tube that matched the solvent.

### 4.8. Anti-Trypanosomal Assay

Initial anti-trypanosomal tests against *T. brucei* were carried out using a resazurin (Alamar blue) cell proliferation assay according to a previously modified protocol [24], adapted from [53]. Using this assay, the half maximal effective concentration (EC_50_) values for compounds were determined, by fitting the dose–response data to a sigmoid curve with variable slope (GraphPad Prism 8). Testing was carried out against a standard drug-sensitive *T. b. brucei* clone, Lister s427 [24], and two derived drug-resistant lines, B48 [26] and ISMR1 [41], to help identify potential cross-resistance with existing drugs. The experiments were performed independently on four different occasions and the EC_50_ values are presented as average (AVG) and standard error of the mean (SEM). Each test compound or extract was serially diluted over 23 well of a white opaque plastic 96-well plate (F Cell Star, Greiner Bio-one GmbH, Frickenhausen, Germany), with the 24th well left for drug-free control. The assay depends on viable cells metabolising the blue non-fluorescent resazurin dye to the pink fluorescent resorufin [54]. The seeding density at the start of the experiment was 2 × 10^4^ cells/well for each strain and the cells were exposed to the test compounds for 48 h at 37 °C/5% CO_2_. The resazurin was then added to the cells and incubated for a further 24 h under the same conditions to allow the viable cells to metabolise the resazurin dye to resorufin. At the end of the 24 h incubation, fluorescence was determined using a FLUOstar Optima (BMG Labtech, Aylesbury, UK) at wavelengths of 544 nm and 620 nm for excitation and emission, respectively.

### 4.9. Strains and Cultures

Bloodstream forms of *T. b. brucei* were grown in standard HMI-9 medium with 10% fetal bovine serum at 37 °C/5% CO_2_, in vented culture flasks, exactly as described) [40]. The standard laboratory strain Lister 427WT was used as drug sensitive standard and the multi-drug resistant clone B48 [26] was used to assess the potential for cross-resistance with the diamidine and melaminophenyl arsenical classes of trypanocides. *T. b. brucei* clone ISMR1 is cross-resistant to isometamidium and pentamidine, and independent of its mitochonrial DNA [41].

### 4.10. Testing against L. mexicana

*L. mexicana* promastigotes were cultured and the miltefosine APC12-resistant *L. mexicana* was strain was selected as described previously [23]. Both cell lines were screened with propolis samples at a starting concentration of 0.125 mg/mL, doubly diluted 11 times across a 96-well plate in triplicate and incubated for 72 h at 25 °C. Wells with no propolis or test compound added were used as control in the experiment. Resazurin was then added to the cells and incubated for a further 24 h under the same conditions to allow the viable cells to metabolise the resazurin dye to resorufin. Fluorescence was detected using the Varioskan Lux plate reader (Thermo, UK) at wavelengths of 560 nm and 590 nm for excitation and emission, respectively. Viability was taken to be proportional to light emitted from for each drug-treated well, and was expressed as a faction of emission from the ‘no drug’ control. IC_50_ values were determined using Grafit 7.0 software.

## 5. Conclusions

A total of 14 compounds were characterised during this study; two of these were in a mixture. None of the compounds were novel, although we cannot find previous reports of the isolation of 4′,7dimethoxy naringenin, 4′,7 dimethoxy kaempferol, or 4′-methoxy kaempferol from propolis. All of the isolated compounds and crude extracts from the propolis samples were tested for cytotoxicity and for activity against different strains of *T. brucei*. None of the purified compounds were more active against *T. brucei* than the crude extracts, suggesting a degree of synergy between the different compounds within the extracts. Both the crude extracts and the isolated compounds were almost equally effective against the standard drug-sensitive strain and the multi-drug resistant clones B48 and ISMR1 (within ~1.5-fold).

A screen of 35 European samples and modelling of the data [23] suggested that galangin and tectochrysin would be the most active individual compounds. Both of these compounds were isolated and tested for anti-parasite activity in the current study, and galangin was one of the more active compounds against both parasite species. However, tectochrysin showed much lower activity. It might be that together these two compounds show some synergy. With regard to future work, there are still many components in temperate propolis to isolate although in this study some of the major components have been characterised. Overall, there is still a great deal to be understood regarding the significance of propolis to the bee, the variations in its constituents and elucidation of the structures and testing of the full range of components within propolis. These studies will allow the drawing up of a shortlist of active anti-parasite compounds from propolis for more in depth studies of their mechanism of action and their in vivo activity.

## Figures and Tables

**Table 1:** Weight of extracts from the propolis samples.

Serial Number	Sample Code	Sample Origin	Weight of Sample (g)	Weight of Ethanol Extract (g)
1	S224	Midlands—UK	26	11.5
2	S225	Essex—UK	33	15
3	D6	Northampton shire —UK	13	7.5
4	D7	Essex—UK	18	10
5	P	Poland	30	16

**Table 2:** EC_50_ (µg/mL) (*n* = 3) of crude extracts of the propolis samples on Tb S427WT and B48 strains of *T. brucei*.

Sample Code	Tb S427WT		B48			
	AVR µg/mL	SEM	AVR µg/mL	SEM	RF	T-TEST
S224	5.28	0.51	4.7	0.31	0.89	0.395
S225	14.04	0.13	10.6	1.64	0.75	0.102
D6	4.49	0.22	3.0	0.20	0.66	0.007
D7	5.97	0.17	4.6	0.26	0.78	0.013
P	4.45	0.08	6.6	0.18	1.49	<0.001
Pentamidine *	0.0027	<0.001	0.6	0.01	226.61	<0.001

* values in µM; T.b. S427 WT = *Trypanosoma brucei brucei* s427 wild type [24]; B48 = A multi-drug resistant strain, which was derived from a *T. b. brucei* adenosine transporter-1 knockout (TbAT1-KO) strain [25] after increasing exposure to pentamidine and lacks both the TbAT1/P2 transporter and the high affinity pentamidine transporter (HAPT), hence becomes highly resistant (>200 fold) to pentamidine [26]. Pentamidine is a standard HAT drug, used as control in this assay. Statistical significance was determined comparing EC_50_ value of the resistant strain with that of the same sample for the control strain s427. RF: resistance factor, being the EC_50_ value for the resistant strain divided by the EC_50_ value for the control (sensitive) strain. Statistical difference between the EC_50_ values of *T. b. brucei* WT and B48 was determined using an unpaired Student’s *t*-test.

**Table 3:** Activity of isolated compounds against *T. b. brucei* strains (*n* = 4).

	TbS427WT		B48				ISMR1			
Compound	AVG µM	SEM µM	AVG µM	SEM µM	RF	*t*-Test	AVG µM	SEM µM	RF	*t*-Test
Tectochrysin	n.a.	n.a.	n.a.	n.a.	--	--	n.a.	n.a.	--	--
Kaempferol	24.0	0.6	30.2	1.8	1.26	0.017	28.4	2.1	1.18	0.095
Pinocembrin	63.8	2.4	83.4	3.5	1.31	0.003	81.0	4.8	1.27	0.019
4′-Methoxykaempferol	15.2	0.4	22.4	0.3	1.48	0.00001	21.1	0.6	1.39	0.00017
Galangin	28.2	1.3	32.2	2.7	1.14	0.23	26.3	1.2	0.93	0.32
Chrysin	69.0	2.0	106	2.6	1.53	0.00003	84.0	6.7	1.22	0.076
Apigenin	25.0	0.5	29.1	0.3	1.17	0.0005	32.3	3.4	1.29	0.076
Pinostrobin	52.9	0.4	56.3	1.6	1.07	0.078	56.7	1.6	1.07	0.054
Cinnamic acid	62.2	2.8	84.6	1.4	1.36	0.0004	64.5	4.3	1.04	0.67
Kaempferol 4′ 7-dimethyl ether	95.2	7.7	103	4.6	1.08	0.40	94.1	8.4	0.99	0.926
Naringenin 4′,7-dimethyl ether	17.5	0.4	21.9	0.3	1.25	0.00012	16.1	0.3	0.92	0.030
Coumaric acid cinnamyl ester and Coumaric acid benzyl ester	45.8	1.0	51.7	0.9	1.13	0.004	52.6	1.6	1.15	0.012
Pentamidine	0.0024	0.0004	0.47	0.04	196	0.00003	0.053	0.002	21.8	<0.0001

n.a. = not active at 370 µM.

**Table 4:** Activity of isolated compounds against *L. mexicana* strains (*n* = 3).

	Leish WT		C12Rx		
Sample	AVG (µM)	SEM (µM)	AVG (µM)	SEM (µM)	RF
Pinobanksin 3-*O*-acetate	163	37	n.t.	n.t.	
Tectochrysin	>400		308	33	
Kaempferol	414	130	n.t.	n.t.	
Pinostrobin	25.1	2.9	6.1	1.0	0.24
4′-methoxykaempferol	41.4	8.6	10.4	0.9	0.25
Galangin	20.2	5.0	54.4	12.7	2.6
Chrysin	17.6	1.7	21.4	3.4	1.25
Apigenin	24.3	3.0	10.3	0.9	0.43
Pinocembrin	60.9	7.8	31.5	4.4	0.52
Cinnamic acid	111	14	34.6	6.8	0.31
Naringenin 4′7- dimethyl ether	17.8	0.9	14.0	1.7	0.79
Coumaric acid cinnamyl/benzyl ester	13.1	1.00	13.6	2.5	1.04
Kaempferol 4′ 7-dimethyl ether	12.9	3.7	n.t.	n.t.	
Miltefosine	4.91	0.14	70.5	3.8	14.4

n.t. = not tested.

**Table 5:** Mobile phase for LC-MS analysis of propolis extracts.

Time	Solvent A (%)	Solvent B (%)	Flow Rate µL/min
0.00	70.0	30.0	300
30.0	0.00	100.0	300
40.0	0.00	100.0	300
41.0	70.0	30.0	300
50.0	70.0	30.0	300
	100.0	0.00	300

Solvent A, 0.1% *v*/*v* formic acid in H_2_O; Solvent B, 0.1 % *v*/*v* formic acid in acetonitrile.

## Data Availability

All the data presented in this study are contained in this article and its Supplementary Materials.

## Supplementary Materials

The following are available online: Figure S1: Chemical structure of Pinobanksin 3-*O*-acetate; Figure S2: Extracted ion chromatogram and the mass spectrum in the negative ion mode (-ve ESI) for Pinobanksin 3-*O*-acetate; Figure S3: ^1^H NMR (400 MHz) of Pinobanksin 3-*O*-acetate in CDCl_3_; Figure S4: ^13^C NMR (400 MHz) of Pinobanksin 3-*O*-acetate in CDCl_3_; Figure S5: Chemical structure of tectochrysin; Figure S6: Extracted ion chromatogram and mass spectrum in the positive ion mode (+ve ESI) for tectochrysin; Figure S7: ^1^H NMR (400 MHz) of tectochrysin in CDCl_3_; Figure S8: ^13^C NMR (400 MHz) of 7-methoxychrysin in CDCl_3_; Figure S9: Chemical structure of Kaempferol; Figure S10: Extracted ion chromatogram and the mass spectrum in the negative ion mode (-ve ESI) for Kaempferol; Figure S11: ^1^H NMR (400 MHz) of Kaempferol in CDCl_3_; Figure S12: ^13^C NMR (400 MHz) of kaempferol in CDCl_3_; Figure S13: Chemical structure of pinocembrin; Figure S14: Extracted ion chromatogram and the mass spectrum in the negative ion mode (-ve ESI) for Pinocembrin; Figure S15: ^1^H NMR (400 MHz) of Pinocembrin in Acetone-d6; Figure S16:^13^C NMR (400 MHz) of Pinocembrin in Acetone-d6; Figure S17: Chemical structure of 4′-methoxykaempferol; Figure S18: Extracted ion chromatogram and the mass spectrum in positive ion mode for 4′-Methoxykaempferol; Figure S19:^1^H NMR (400 MHz) of 4′-Methoxykaempferol in Acetone d6; Figure S20:^13^C NMR (400 MHz) of 4′-Methoxykaempferol in Acetone d6; Figure S21: Chemical structure of Galangin; Figure S22: Extracted ion chromatogram and the mass spectrum in the negative ion mode for Galangin; Figure S23:^1^H NMR (400 MHz) of Galangin in Acetone d6; Figure S24:^13^C NMR (400 MHz) of Galangin in Acetone d6; Figure S25: Chemical structure of Chrysin; Figure S26: Extracted ion chromatogram and mass spectrum in positive ion mode for Chrysin; Figure S27: ^1^H NMR (400 MHz) of Chrysin in DMSO d6; Figure S28:^13^C NMR (400 MHz) of Chrysin in DMSO d6; Figure S29: Chemical structure of Apigenin; Figure S30: Extracted ion chromatogram and mass spectrum in negative ion mode for Apigenin; Figure S31: ^1^H NMR (400 MHz) of Apigenin in Acetone d6; Figure S32: ^13^C NMR (400 MHz) of Apigenin in Acetone d6; Figure S33: Chemical structure of Pinostrobin; Figure S34: Extracted ion chromatogram and mass spectrum in positive ion mode for Pinostrobin; Figure S35: ^1^H NMR (400 MHz) of Pinostrobin in CDCl_3_; Figure S36: ^13^C NMR (400 MHz) of Pinostrobin in CDCl_3_; Figure S37: Chemical structure of Cinnamic acid; Figure S38: Extracted ion chromatogram and mass spectrum in positive ion mode for Cinnamic acid; Figure S39: ^1^H NMR (400 MHz) of Cinnamic acid in CDCl_3_; Figure S40: ^13^C NMR (400 MHz) of Cinnamic acid in CDCl_3_; Figure S41: Chemical structure of Coumaric acid cinnamyl ester; Figure S42: Extracted ion chromatogram and the mass spectrum in the negative ion mode for Coumaric acid cinnamyl ester; Figure S43: ^1^H NMR (400 MHz) of Coumaric acid cinnamyl ester in CDCl_3_; Figure S44: ^13^C NMR (400 MHz) of Coumaric acid cinnamyl ester in CDCl_3_; Figure S45: Chemical structure of Coumaric acid benzyl ester; Figure S46: Extracted ion chromatogram and mass spectrum in positive ion mode for Benzyl *p*-coumarate; Figure S47: ^1^H NMR (400 MHz) of Coumaric acid benzyl ester in CDCl_3_; Figure S48: ^13^C NMR (400 MHz) of Coumaric acid benzyl ester in CDCl_3_; Figure S49: Chemical structure of 4′,7-dimethoxykaempferol; Figure S50: Extracted ion chromatogram and mass spectrum in positive ion mode for 4′,7-Dimethoxykaempferol; Figure S51: ^1^H NMR (400 MHz) of 4′,7-Dimethoxykaempferol in CDCl_3_; Figure S52: ^13^C NMR (400 MHz) of 4′,7-Dimethoxykaempferol in CDCl_3_; Figure S53: ^1^H NMR (400 MHz) of Naringenin 4′,7-dimethyl ether in CDCl_3_; Figure S54: ^13^C NMR (400 MHz) of Naringenin 4′,7-dimethyl ether in CDCl_3_; Table S1: Chemical shifts for Pinobanksin 3-*O*-acetate; Table S2: Chemical shifts for Tectochrysin (7-*O*-Methoxychrysin); TableS3: Chemical shifts for Kaempferol; Table S4: Chemical shifts for Pinocembrin (400 MHz) in Acetone d6; Table S5: Chemical shifts for 4′-Methoxykaempferol; Table S6: Chemical shifts for Galangin; Table S7: Chemical shifts for Chrysin; Table S8: Chemical shifts for Apigenin; Table S9: Chemical shifts for pinostrobin; Table S10: Chemical shifts for Cinnamic acid; Table S11: Chemical shifts for Coumaric acid cinnamyl ester; Table S12: Chemical shifts for Coumaric acid benzyl ester; Table S13: Chemical shifts for 4′,7-Dimethoxykaempferol; Table S14: Chemical shifts for Naringenin 4′,7-dimethyl ether.

## Sample Availability

The exact propolis samples were unique even though representative of the general location from where they were derived. Samples of any of the isolated the compounds may be available from the authors but may require re-purification.
