# Supplementary material for: Activity of Compounds from Temperate Propolis against Trypanosoma brucei and Leishmania mexicana
Source: Molecules. 2021 Jun 26;26(13):3912. doi: 10.3390/molecules26133912 (PMC8272135; doi:10.3390/molecules26133912)
Supplement: Supplementary file 1 [file molecules-26-03912-s001.zip › molecules-1271651-SI.pdf]

molecules

MDPI

Article

Activity of Compounds from European Propolis against *Trypanosoma brucei* and *Leishmania mexicana*.

Abdullah Alotaibi[1], Godwin U. Ebiloma[2,3], Roderick Williams[4], Ibrahim A. Alfayez,[2,5] Manal J. Natto,[2] Sameah Alenezi[1], Weam Siheri[6] Malik AlQarni[7], John O. Igoli[1,2,8], James Fearnley[9], Harry P. de Koning[2,*] and David G. Watson[1*]

1. University of Strathclyde, Strathclyde Institute of Pharmacy and Biomedical Science, 161 Cathedral Street, Glasgow, G4 0RE, UK.
2. Institute of Infection, Immunity and Inflammation, College of Medical, Veterinary and Life Sciences, University of Glasgow, Glasgow G12 8TA, UK
3. School of Health and Life Sciences, Teesside University, Middlesbrough, United Kingdom
4. IBEHR, School of Health and Life Science, University of the West of Scotland, High Street, Paisley PA1 2BE
5. Qassim Health Cluster, Ministry of Health, Buraydah 52367, Saudi Arabia Department of Pharmaceutical Chemistry, College of Clinical Pharmacy, Imam Abdulrahman Bin Faisal University, Dammam 31441, Saudi Arabia.
6. Department of Pharmacognosy and Natural Products ,Faculty of Pharmacy, University of Tripoli, Tripoli , Libya.
7. Department of Pharmaceutical Chemistry, College of Clinical Pharmacy, Imam Abdulrahman Bin Faisal University, Dammam 31441, Saudi Arabia.
8. Department of Chemistry, University of Agriculture, PMB 2373, Makurdi, Nigeria
9. BeeVital, Whitby, North Yorkshire, YO22 5JR, UK.

* Correspondence: Correspondence: harry.de-koning@glasgow.ac.uk or d.g.watson@strath.ac.uk

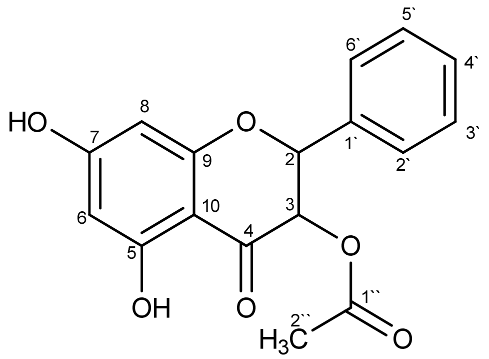

Figure S1. Chemical structure of Pinobanksin 3-O-acetate.

Table S1. Chemical shifts for Pinobanksin 3-O-acetate.

Position	Experimental		Literature*	
	^{1}H δ ppm (mult, J in Hz)	^{13}C δ ppm, (mult)	^{1}H δ ppm, (mult, J in Hz)	^{13}C δ ppm, (mult)
1	-	-	-	-
2	5.36 (1H, d, 11.7)	81.4 (CH)	5.36 (1H, d, *J* = 11.7)	81.3
3	5.85 (1H, d, 11.7)	72.6 (CH)	5.81 (1H, d, *J* = 11.7)	72.4
4	-	191.7 (C)	-	191.6
5	-	163.6 (C)	-	162.5
6	6.01 (1H, d, 2.2)	96.0 (CH)	6.00 (1H, d, *J* = 2.2)	95.9
7	-	166.9 (CH)	-	165.2
8	6.06 (1H, d, 2.2)	97.36 (C)	6.04 (1H, d, *J* = 2.2)	97.4
9	-	165.8 (C)	-	164.2
10	-	102.0 (C)	-	101.9
1`	-	135.9 (C)	-	135.1
2`	7.49(m)	127.4 (CH)		127.3
3`	7.44(m)	128.8 (CH)		128.7
4`	7.45(m)	129.6 (CH)	7.44 (5H, m)	129.6
5`	7.44(m)	128.8 (CH)		128.7
6`	7.49(m)	127.4 (CH)		127.3
1``	-	169.7 (C)	-	169.5
2``	2.06(m)	20.47 (CH_3)	2.02 (3H, s)	20.3
5-OH	11.51 (s)	-	11.47 (1H, s)	-

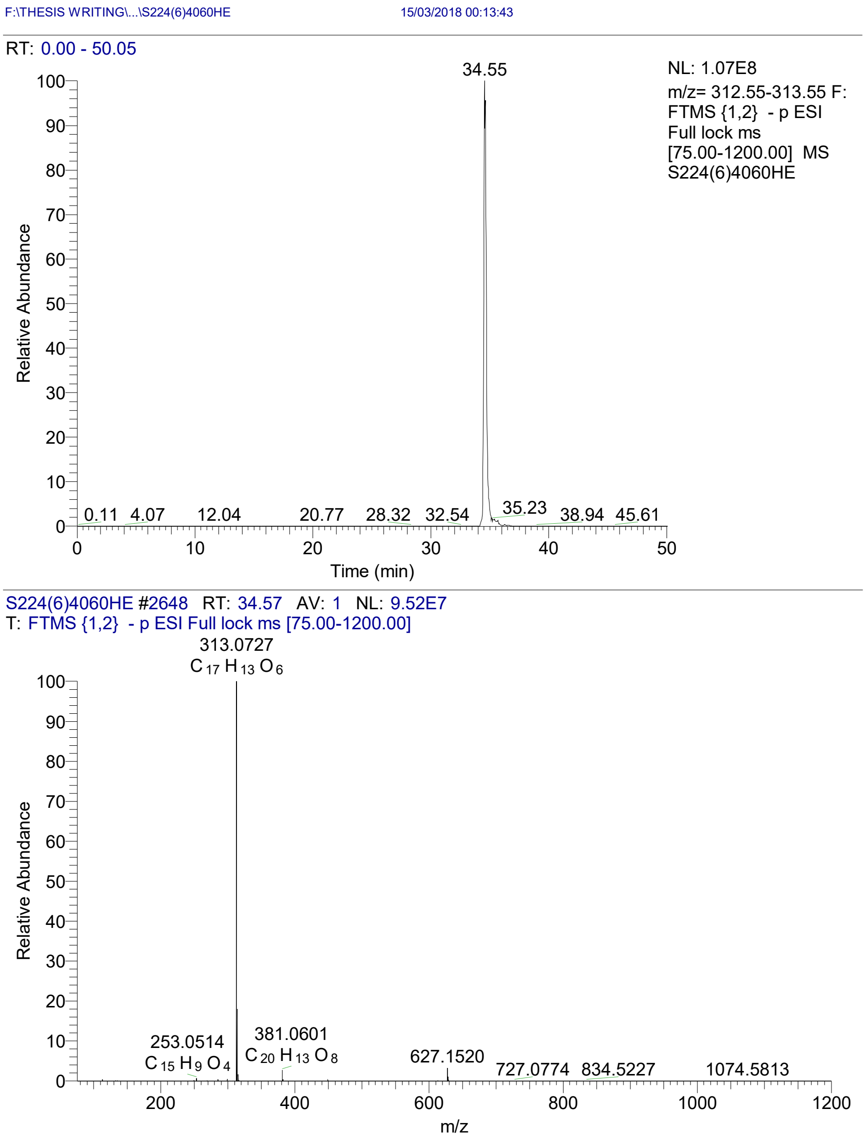

Figure S2. Extracted ion chromatogram and the mass spectrum in the negative ion mode (-ve ESI) for Pinobanksin 3-O-acetate.

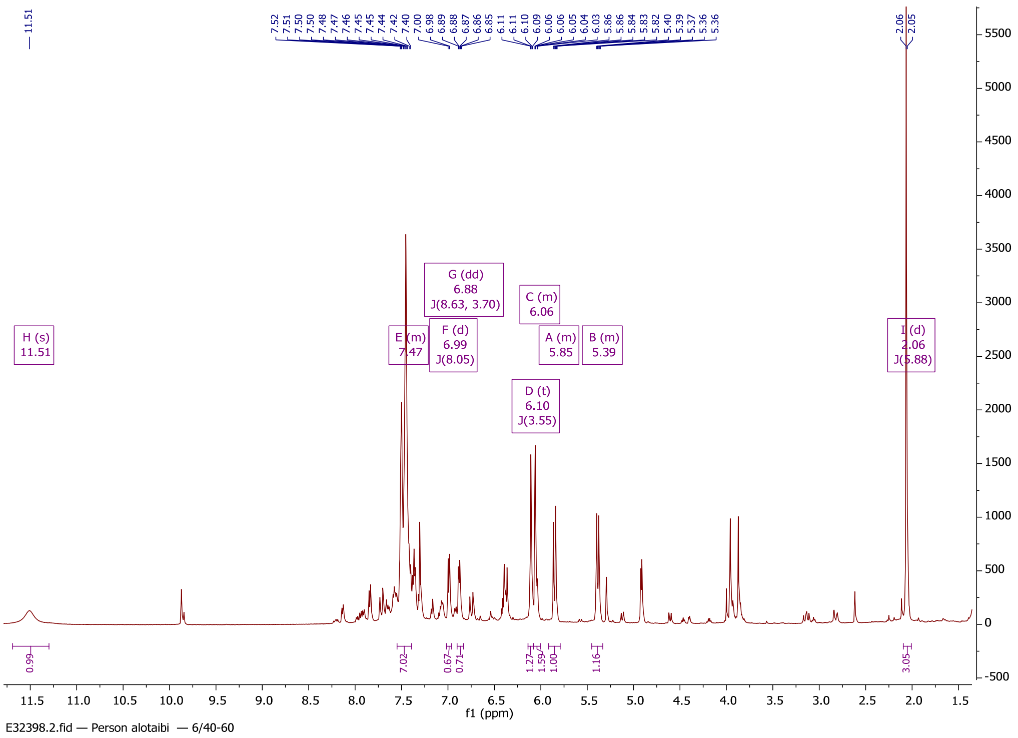

Figure S3. ^{1}H NMR (400 MHz) of Pinobanksin 3-O-acetate in CDCl3.

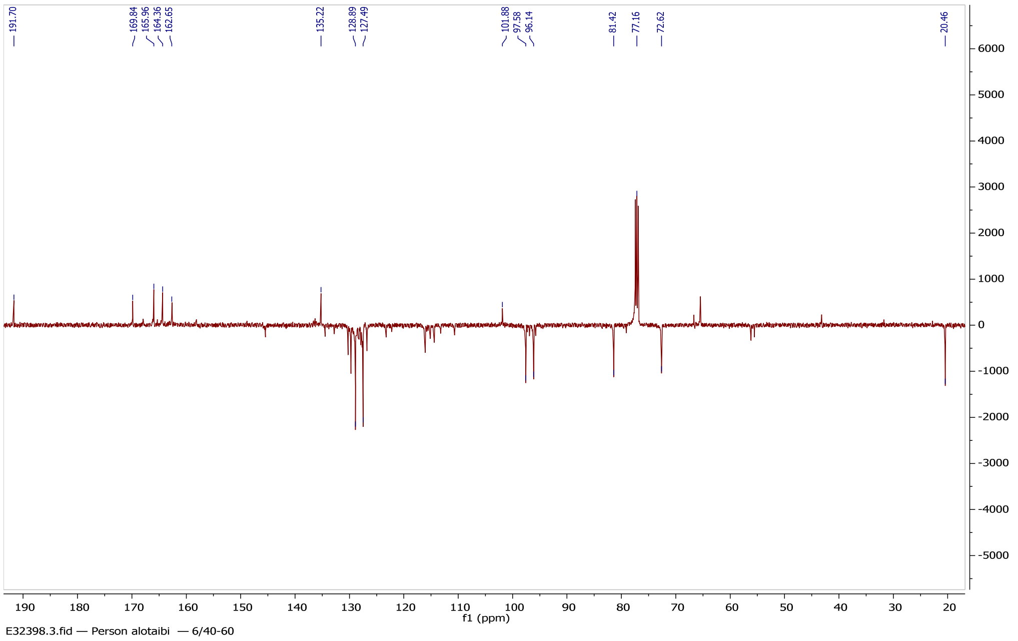

Figure S4. ^{13}C NMR (400 MHz) of Pinobanksin 3-O-acetate in $CDCl_3$.

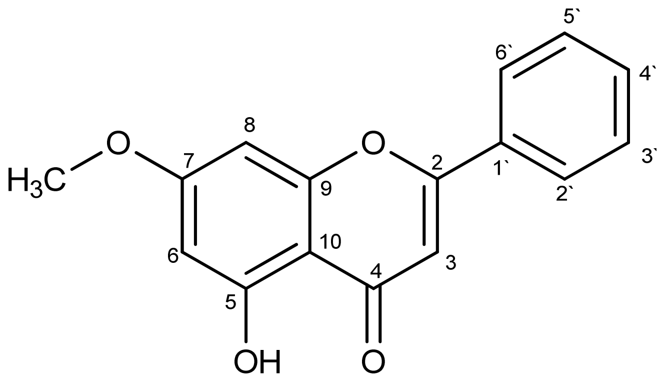

Figure S5. Chemical structure of 7-O-methoxychrysin.

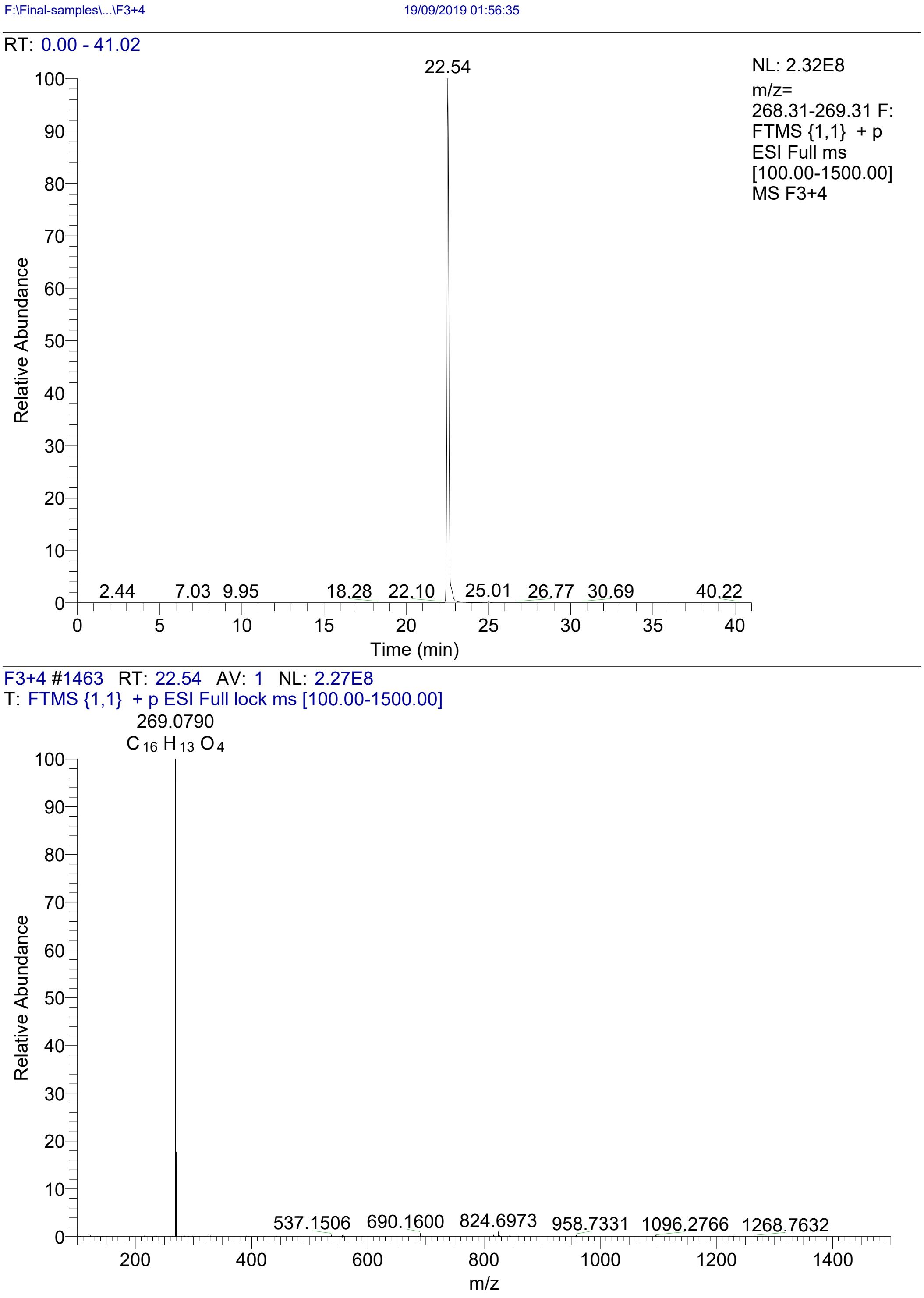

Figure S6. Extracted ion chromatogram and mass spectrum in the positive ion mode (+ve ESI) for 7-methoxychrysin.

Table S2. Chemical shifts for 7-O-Methoxy Chrysin.

Position	7-O-Methoxy Chrysin		Literature*	
	^{1}H δ ppm (mult, J in Hz)	^{13}C δ ppm, (mult)	^{1}H δ ppm, (mult, *J* in Hz)	^{13}C δ ppm, (mult)
1	-	-	-	-
2	-	164.6 (C)	-	164.0
3	6.70 (1H, s)	105.6 (CH)	6.67(s)	105.9
4	-	182.9(C)	-	182.5
5	-	162.8 (C)	-	162.2
6	6.41 (1H, d, 2.2)	98.1 (CH)	6.39(s)	98.2
7	-	165.8 (C)	-	165.6
8	6.54 (1H, d, 2.2)	92.6 (CH)	6.51(s)	92.7
9	-	156.0 (CH)	-	157.8
10	-	105.9	-	105
1`	-	132.6 (C)	-	131.9
2`	7.90 (1H, d)	126.4 (CH)	7.89(d,7.8)	126.3
3`	7.54 (1H, m)	129.0(CH)	7.54 (m)	129.1
4`	7.54 (1H, m)	131.0 (CH)	7.52 (m)	129.1
5`	7.54 (1H, m)	129.0 (CH)	7.54 (m)	129.1
6`	7.90 (1H, d)	126.4 (CH)	7.89(d,7.8)	126.3
7-OCH_3	3.90 (3H, s)	55.4 (CH)	3.89(s)	55.4
5-OH	12.72 (s)	-	12.73(s)	-

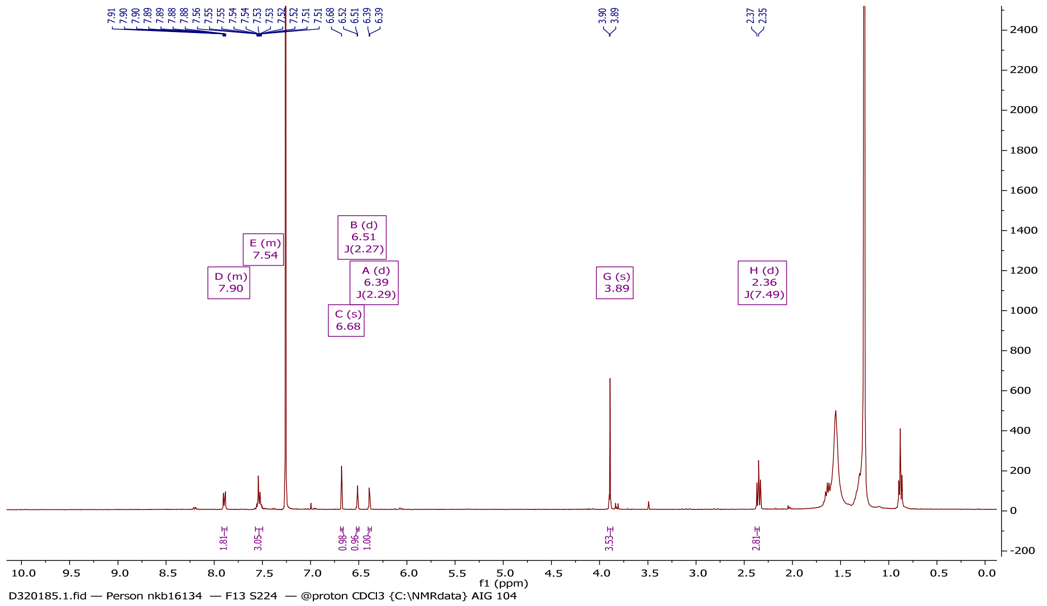

Figure S7. ^{1}H NMR (400 MHz) of 7-methoxychrysin in $CDCl_3$.

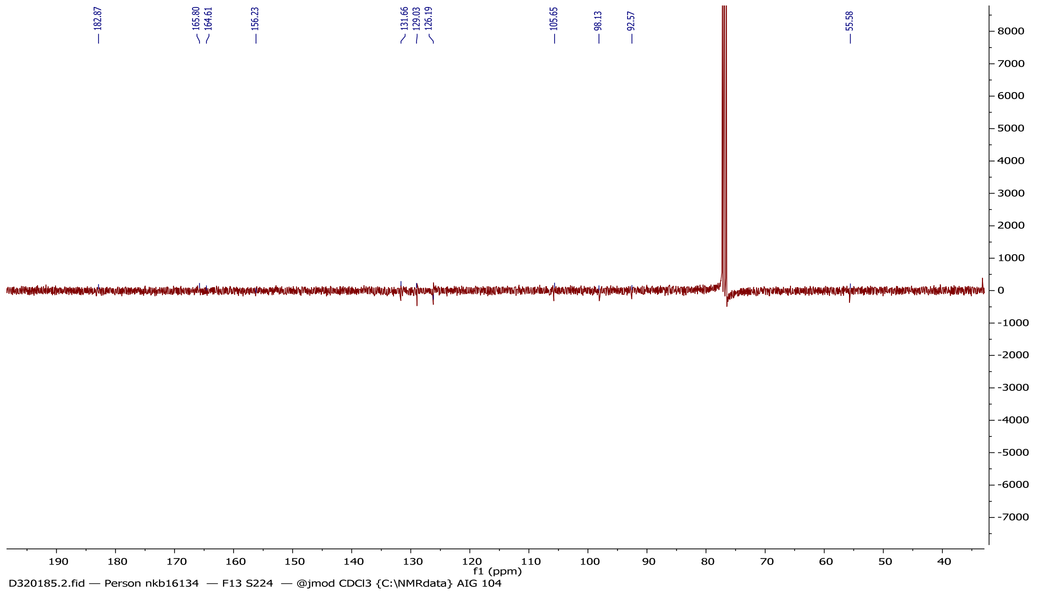

Figure S8. ^{13}C NMR (400 MHz) of 7-methoxychrysin in $CDCl_3$.

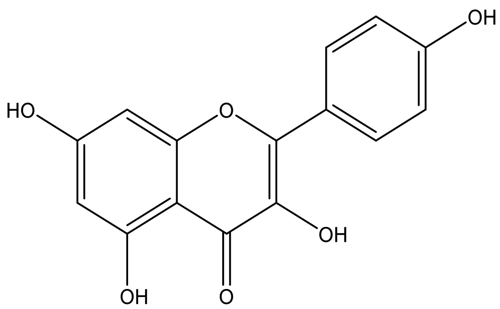

Figure S9. Chemical structure of Kaempferol.

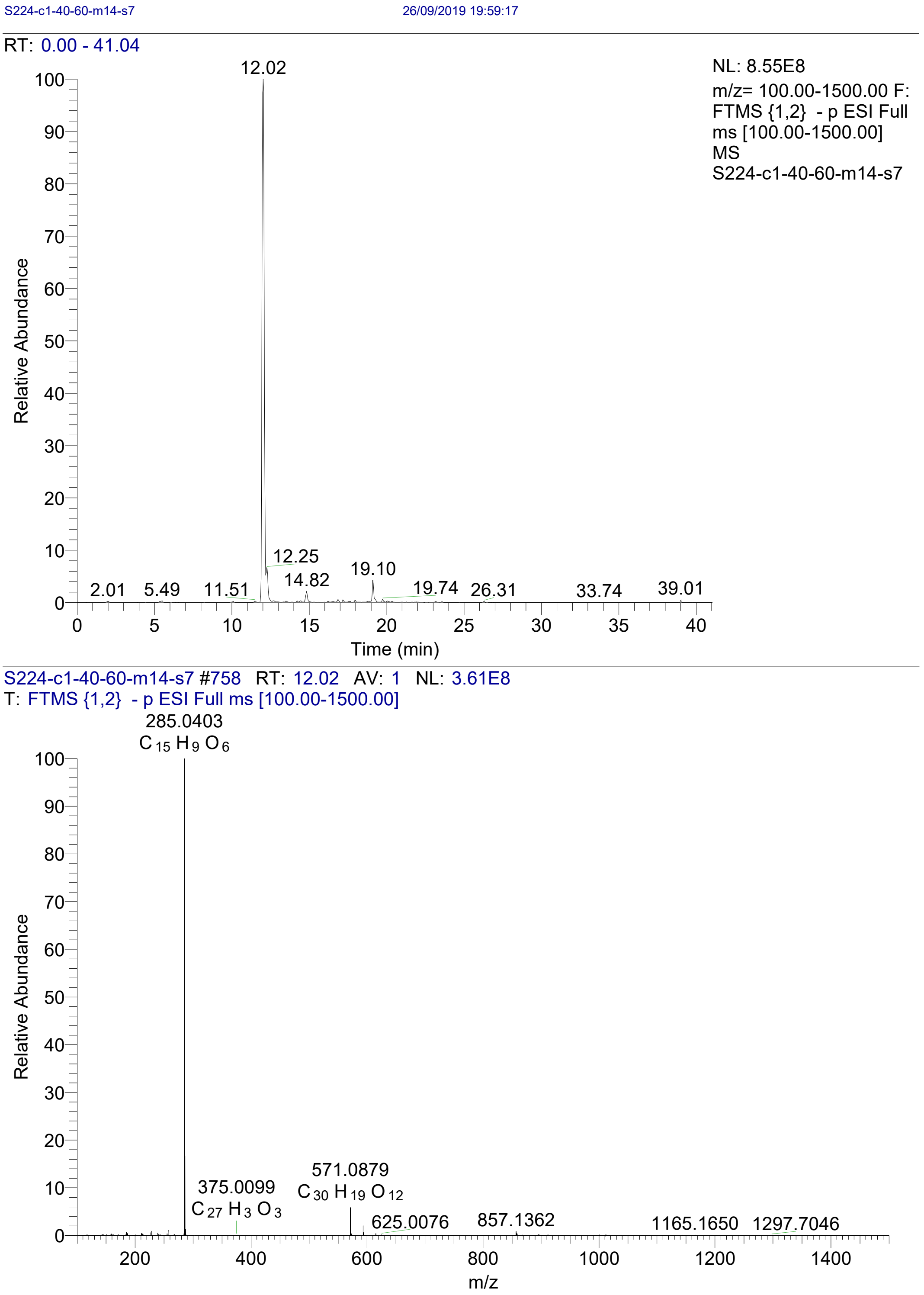

Figure S10. Extracted ion chromatogram and the mass spectrum in the negative ion mode (-ve ESI) for Kaempferol.

TableS3. Chemical shifts for Kaempferol.

Position	**Kaempferol**		Literature*	
	^{1}H δ ppm (mult, *J* in Hz)	^{13}C δ ppm, (mult)	^{1}H δ ppm, (mult, *J* in Hz)	^{13}C δ ppm, (mult)
1	-	-	-	-
2	-	146.9 (C)	-	146.7
3	-	137.1	-	135.5
4	-	175.3 (C)	-	175.8
5	-	160.6 (C)	-	160.6
6	6.27 (1H, d, 2.2)	98.3 (C)	6.21 (1H, d,)	98.1
7	-	163.6 (C)	-	163.8
8	6.54 (1H, d, 2.2)	93.7 (CH)	6.46 (1H, d,)	93.4
9	-	156.6 (C)	-	156.1
10	-	103.5 (C)	-	103.0
1`	-	122.2(C)	-	121.6
2`	8.16 (1H, m)	129.7 (CH)	8.07 (1H, dd)	129.5
3`	7.02 (1H, m)	115.9 (CH)	6.94 (1H, dd)	115.4
4`	-	159.6 (C)		159.1
5`	7.02 (1H, m)	115.9 (CH)	6.94(dd)	115.4
6`	8.16 (1H, m)	129.7 (CH)	8.07(dd)	129.5
7-OH	-	-	10.78(s)	-
5-OH	12.16 (s)	-	12.50 (1H, s)	-
3-OH		-	9.35(s)	
4`-OH	-			

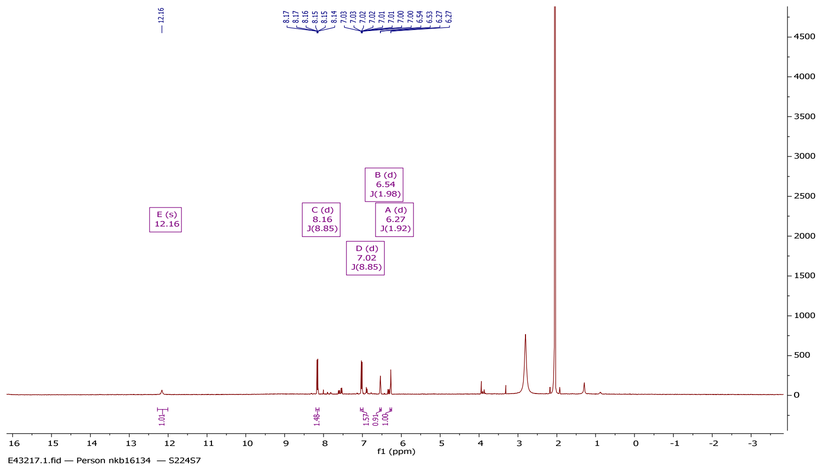

Figure S11. ^{1}H NMR (400 MHz) of Kaempferol in $CDCl_3$.

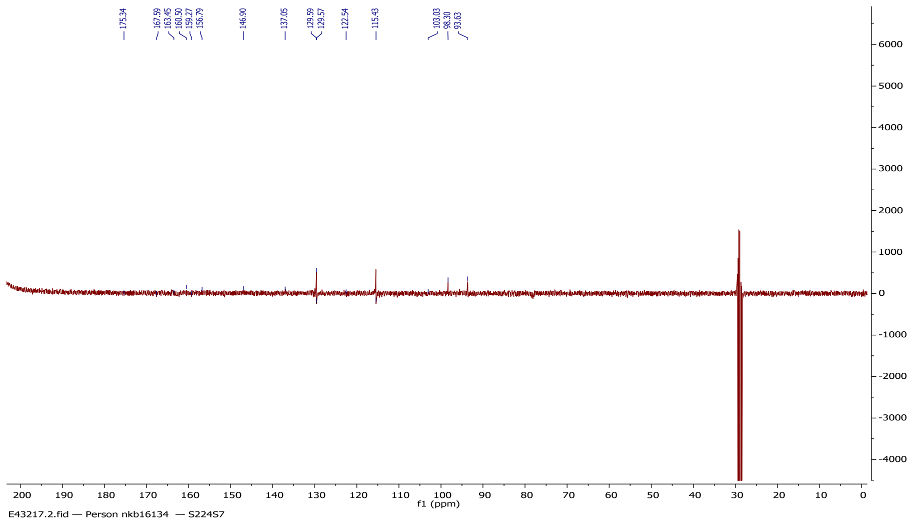

Figure S12. ^{13}C NMR (400 MHz) of Kaempferol in $CDCl_3$.

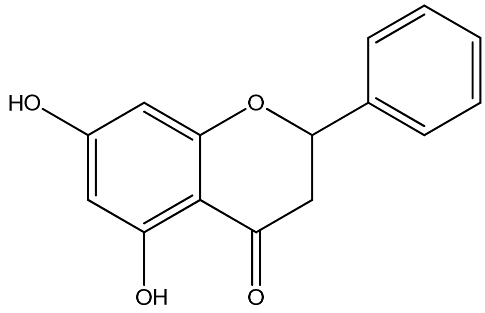

Figure S13. Chemical structure of Pinocembrin.

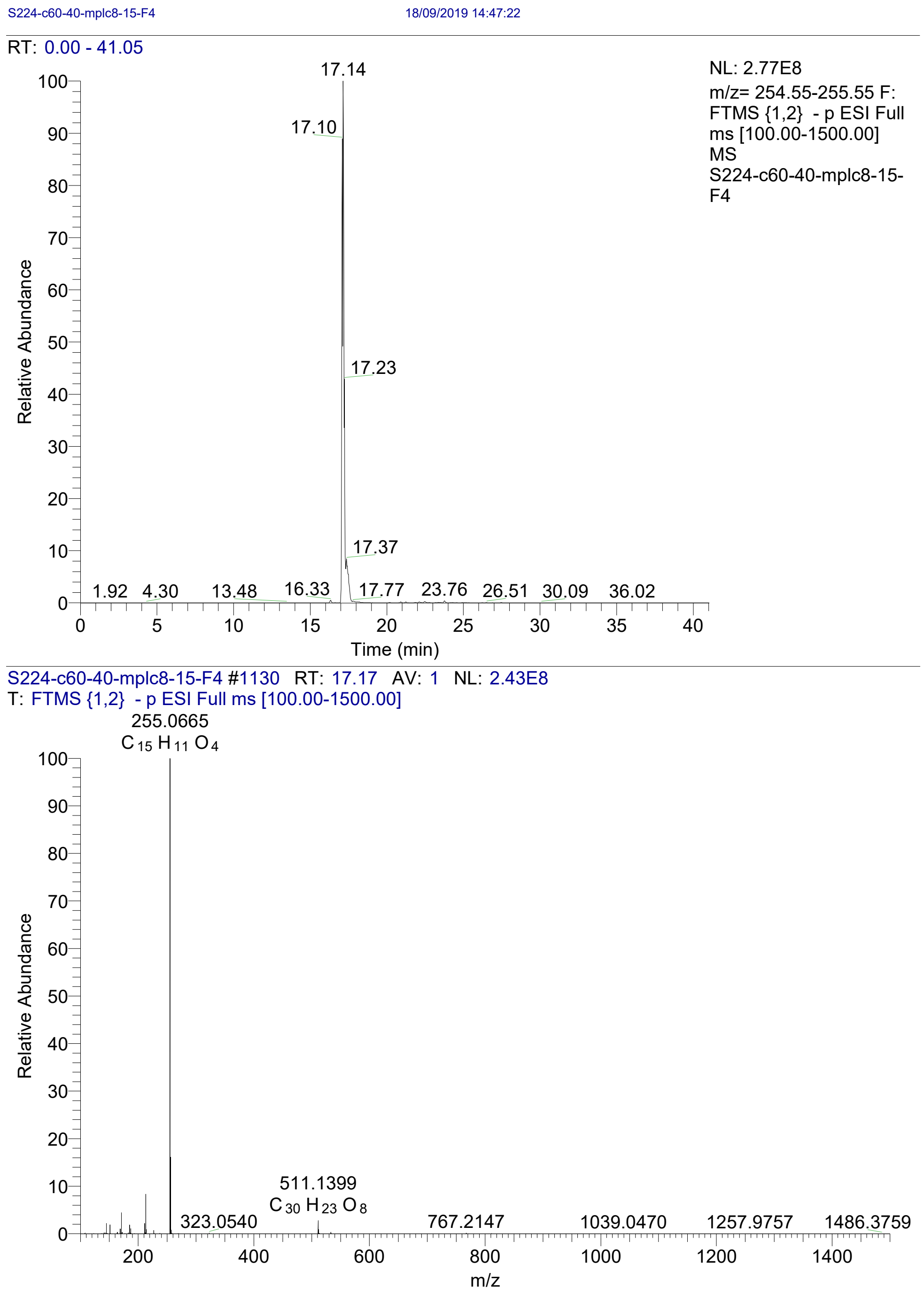

Figure S14. Extracted ion chromatogram and the mass spectrum in the negative ion mode (-ve ESI) for Pinocembrin.

Table S4. Chemical shifts for Pinocembrin (400 MHz) in Acetone d6

Position	**Pinocembrin**		Literature*	
	^{1}H δ ppm (mult, *J* in Hz)	^{13}C δ ppm, (mult)	^{1}H δ ppm, (mult, J in Hz)	^{13}C δ ppm, (mult)
1			-	-
2	5.58(1H, dd,12.8,3.2)	78.1(CH)	5.58 (1H, dd)	78.21
3a	3.17(1H, dd,17.1,12.8)	43.7 (CH_2)	3.23 (1H, dd)	42.77
3b	2.82(1H, dd)		2.79(1H, dd)	
4	-	196.8(C)		196.45
5	-	164.2(C)	-	164.10
6	5.97 (1H, d, 2.2)	96.0 (C)	5.87(dd)	96.50
7	-	166.2(C)	6.00 (1H, d, *J* = 2.2)	167.23
8	6.01 (1H, d, 2.1)	95.0 (CH)	5.87(dd)	95.62
9	-	162.9 (C)	-	163.65
10	-	102.2 (C)	-	102.30
1`	-	139.1(C)	-	139.64
2`	7.57 (1H, m)	127.3 (CH)	7.52(d)-	127.12
3`	7.45 (1H, m)	129.5 (CH)	7.34(5H, m)	129.13
4`	7.40 (1H, m)	129.4 (CH)	7.34(5H, m)	129.13
5`	7.45 (1H, m)	129.5 (CH)	7.34(5H, m)	129.13
6`	7.57 (1H, m)	127.3 (CH)	7.52 (5H, m)	127.12
7-OH	10.81 (1H, s)	-	10.79(s)	-
5-OH	12.16(s)	-	12.13(s)	-

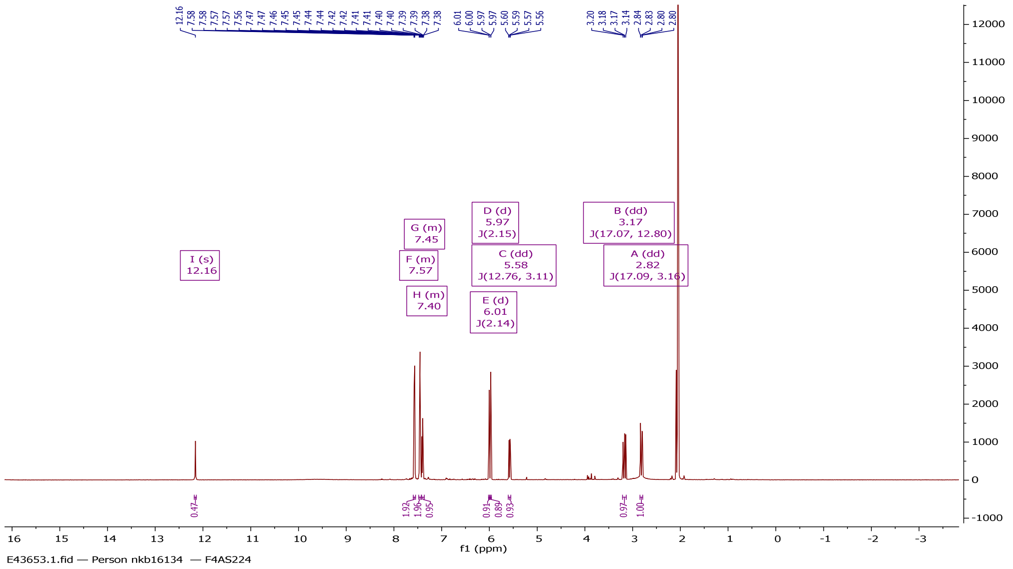

Figure S15. ^{1}H NMR (400 MHz) of Pinocembrin in Acetone-d6.

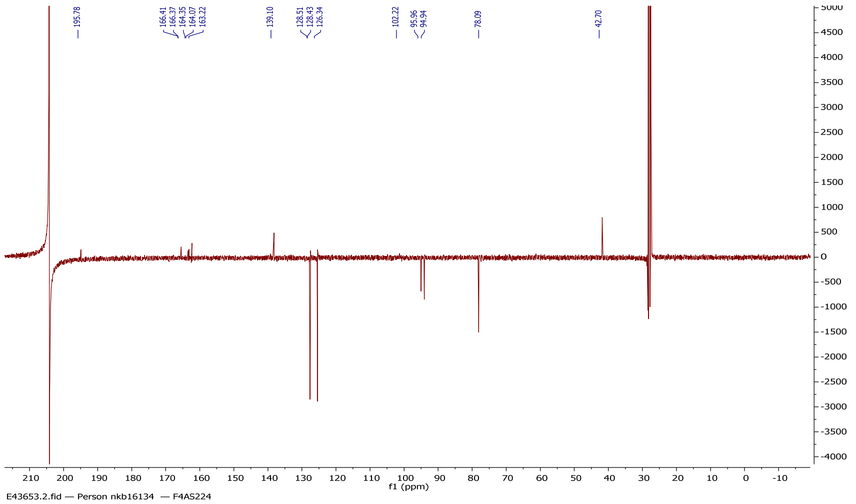

Figure S16. ^{13}C NMR (400 MHz) of Pinocembrin in Acetone-d6.

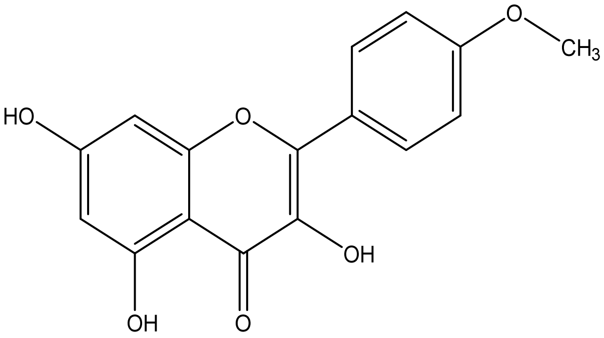

Figure S17. Chemical structure of 4′-methoxykaempferol.

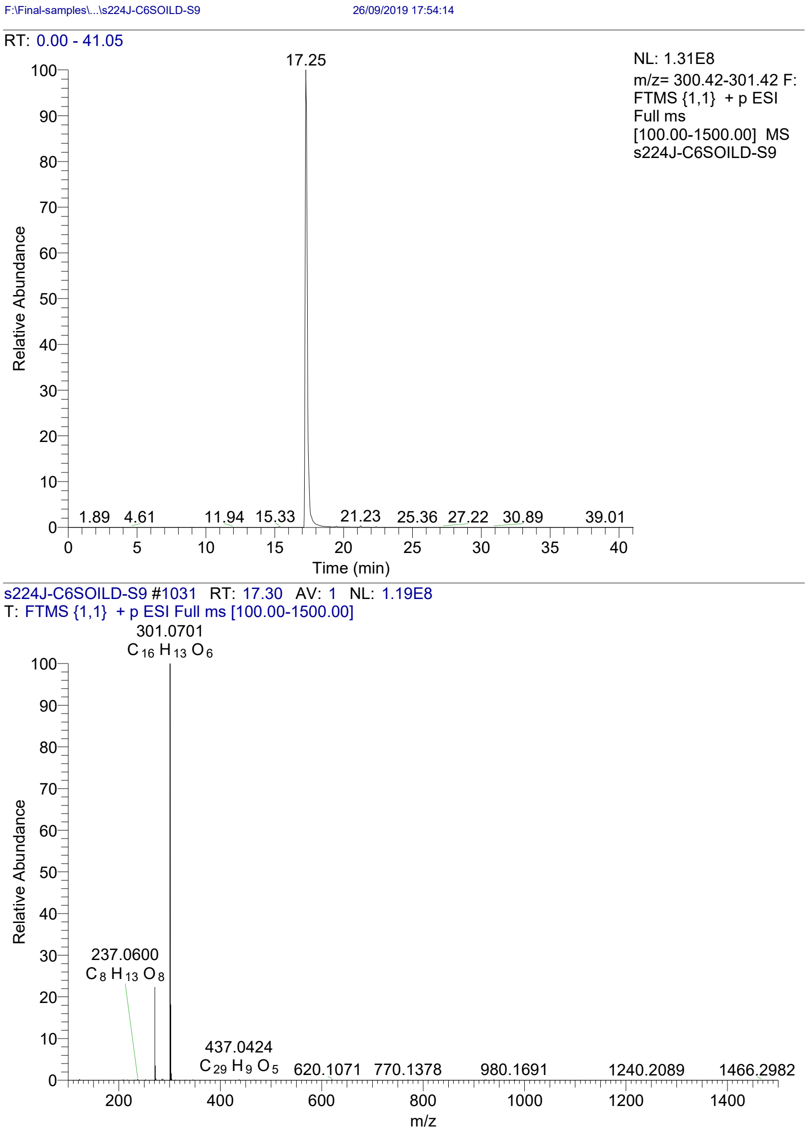

Figure S18. Extracted ion chromatogram and the mass spectrum in positive ion mode for 4'-Methoxykaempferol.

Table S5. Chemical shifts for 4'-Methoxykaempferol.

Position	**5,7-Dihydroxy-4'-methoxyflavonol** (4'-Methoxykaempferol)		Literature*	
	[1]H δ ppm (mult, *J* in Hz)	[13]C δ ppm, (mult)	[1]H δ ppm, (mult, *J* in Hz)	[13]C δ ppm, (mult)
1	-	-	-	-
2	-	146.0(C)	-	146.3
3	-	136.0 (C)	-	136.1
4	-	175.1(C)	-	176.1
5	-	161.5(C)	-	160.8
6	6.29 (1H, d, 2.2)	98.3 (C)	6.29 (1H, d)	98.3
7	-	164.4(C)	-	164.1
8	6.56 (1H, d, 2.2)	93.7 (CH)	6.45 (1H, d,)	93.6
9	-	157.2 (CH)	-	156.3
10	-	103.2(C)	-	103.7
1`	-	122.3(C)	-	123.3
2`	8.23(1H, m)	129.5 (CH)	8.12(1H, d)	129.4
3`	7.14 (1H, m)	113.9 (CH)	7.09(1H, d)	114.1
4`	-	161.5 (C)	-	160.6
5`	7.14 (1H, m)	113.9 (CH)	7.09(1H, d)	114.1
6`	8.23(1H, m)	129.5(CH)	8.12(IH, d)	129.4
7-OH	-	-	10.83 (1H, s)	-
5-OH	12.16(s)	-	12.43 (1H, s)	-
3-OH	-	-	9.47(1H, s)	
4'-OCH_3	3.90 (s)	54.9 (CH_3)	3.81(3H,s)	55.4

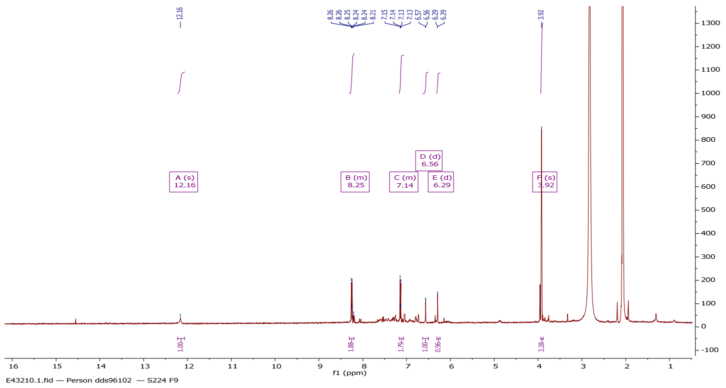

Figure S19. ^{1}H NMR (400 MHz) of 4'-Methoxykaempferol in Acetone d6.

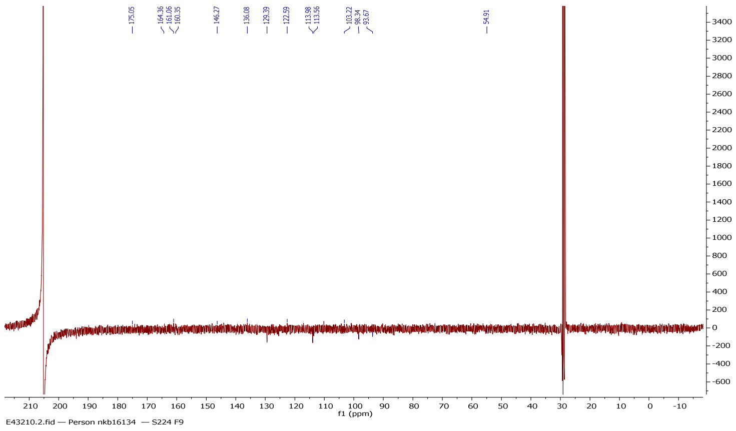

Figure S20. 13C NMR (400 MHz) of 4'-Methoxykaempferol in Acetone d6.

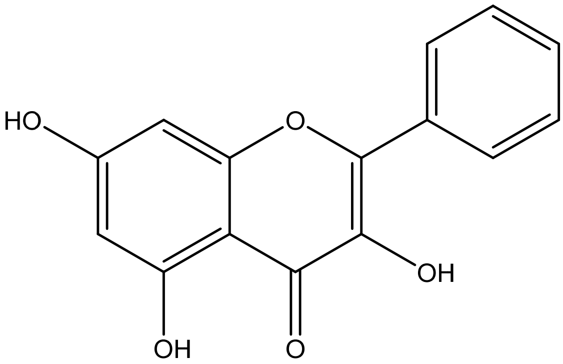

Figure S21. Chemical structure of Galangin.

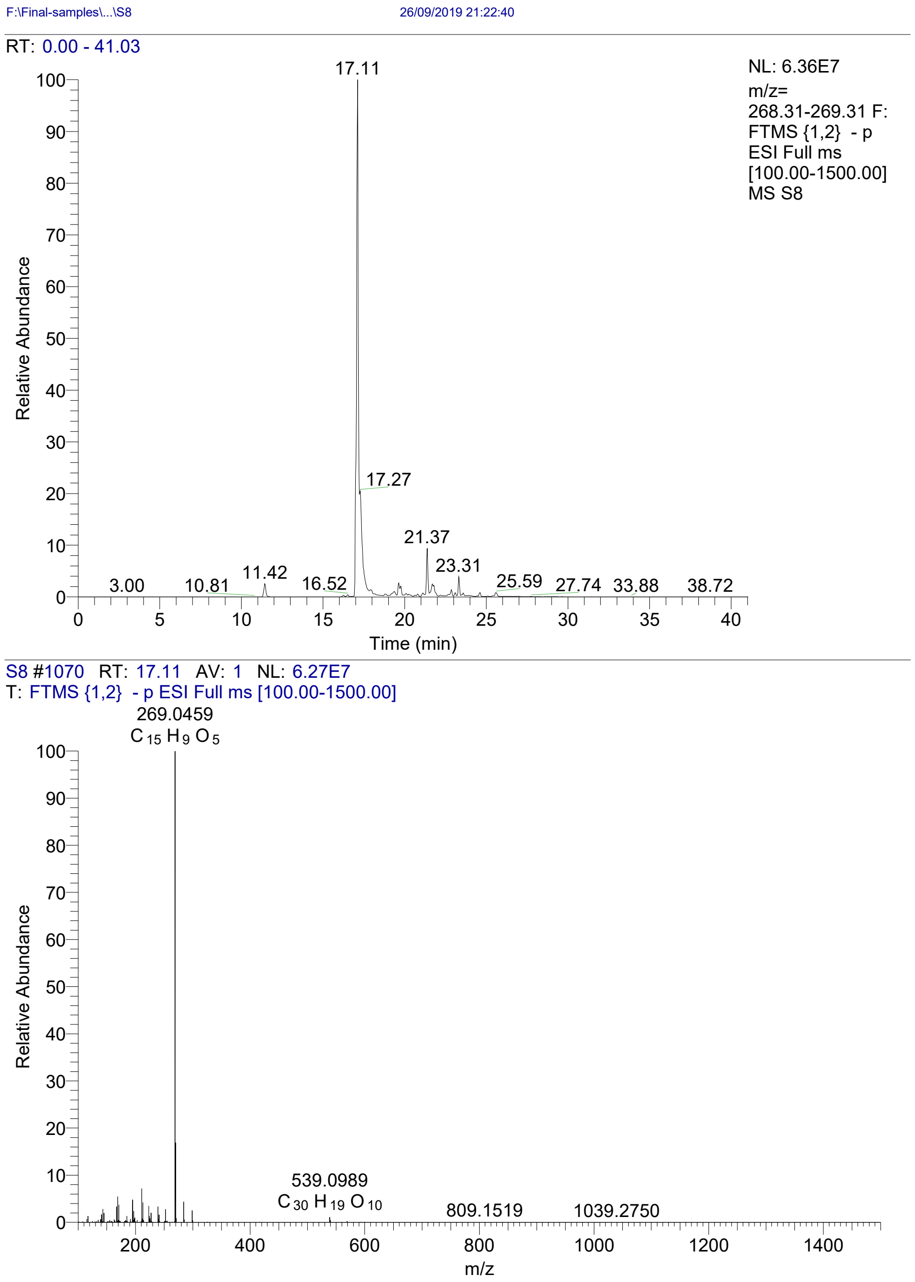

Figure S22. Extracted ion chromatogram and the mass spectrum in the negative ion mode for Galangin.

Table S6. Chemical shifts for Galangin.

Position	**Galangin**		Literature*	
	[1]H δ ppm (mult, J in Hz)	[13]C δ ppm, (mult)	[1]H δ ppm, (mult, J in Hz)	[13]C δ ppm, (mult)
1	-	-	-	-
2	-	145.3 (C)	-	146.11
3	-	137.0 (C)	-	137.52
4	-	176.1(C)		176.68
5	-	161.5(C)	-	169.19
6	6.29 (1H, d, 2.2)	98.4 (C)	6.16(1H, d)	98.74
7	-	164.4(C)	-	164.65
8	6.56 (1H, d, 2.2)	93.7 (CH)	6.40(1H, d)	93.99
9	-	157.2 (CH)	6.04 (1H, d, *J* = 2.2)	156.83
10	-	103.4 (C)	-	103.65
1`	-	131.1(C)	-	131.38
2`	8.26 (1H, m)	127.6 (CH)	8.08 (1H, m)	127.94
3`	7.58 (1H, m)	128.5 (CH)	7.44(1H, m)	128.88
4`	7.52 (1H, m)	130.0 (CH)	7.44 (1H, m)	128.88
5`	7.58 (1H, m)	128.5 (CH)	7.44(1H, m)	128.88
6`	8.26 (1H, m)	127.6 (CH)	8.08 (1H, m)	127.94
7-OH	-	-	10.59(1H, s)	-
5-OH	12.08(s)	-	12.31(1H, s)	-
3-OH	-	-	9.59 (1H, s)	-

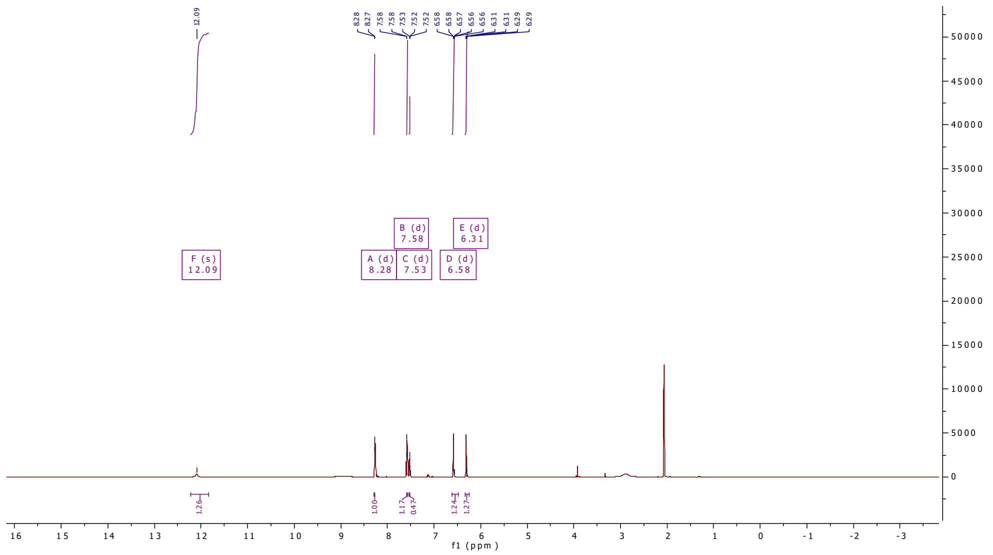

Figure S23. ^{1}H NMR (400 MHz) of Galangin in Acetone d6.

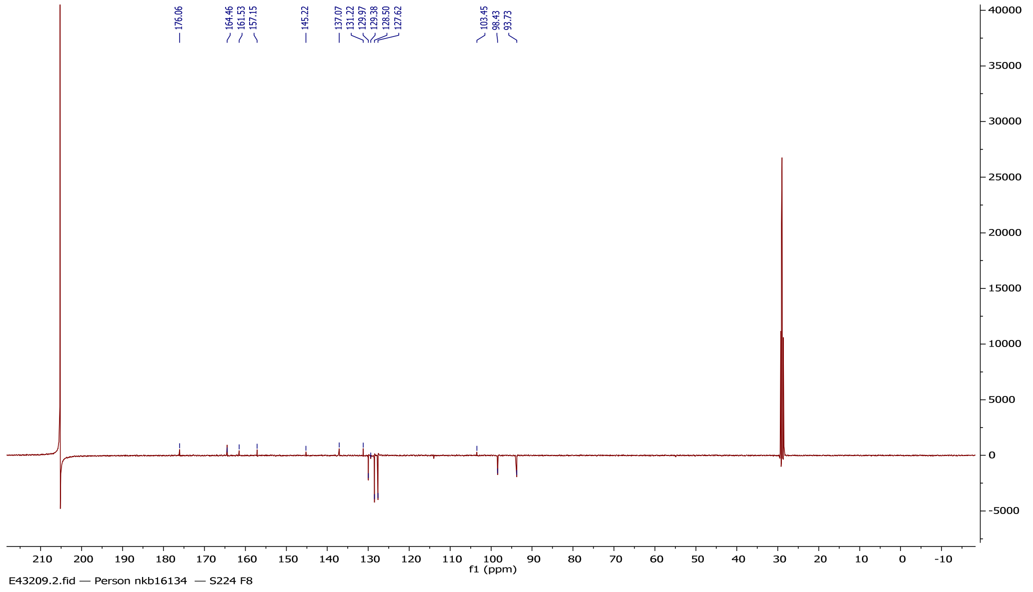

Figure S24.^{13}C NMR (400 MHz) of Galangin in Acetone d6.

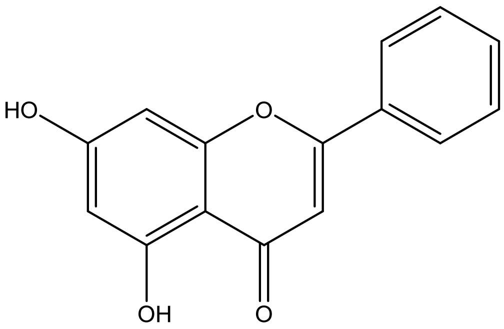

Figure S25. Chemical structure of Chrysin.

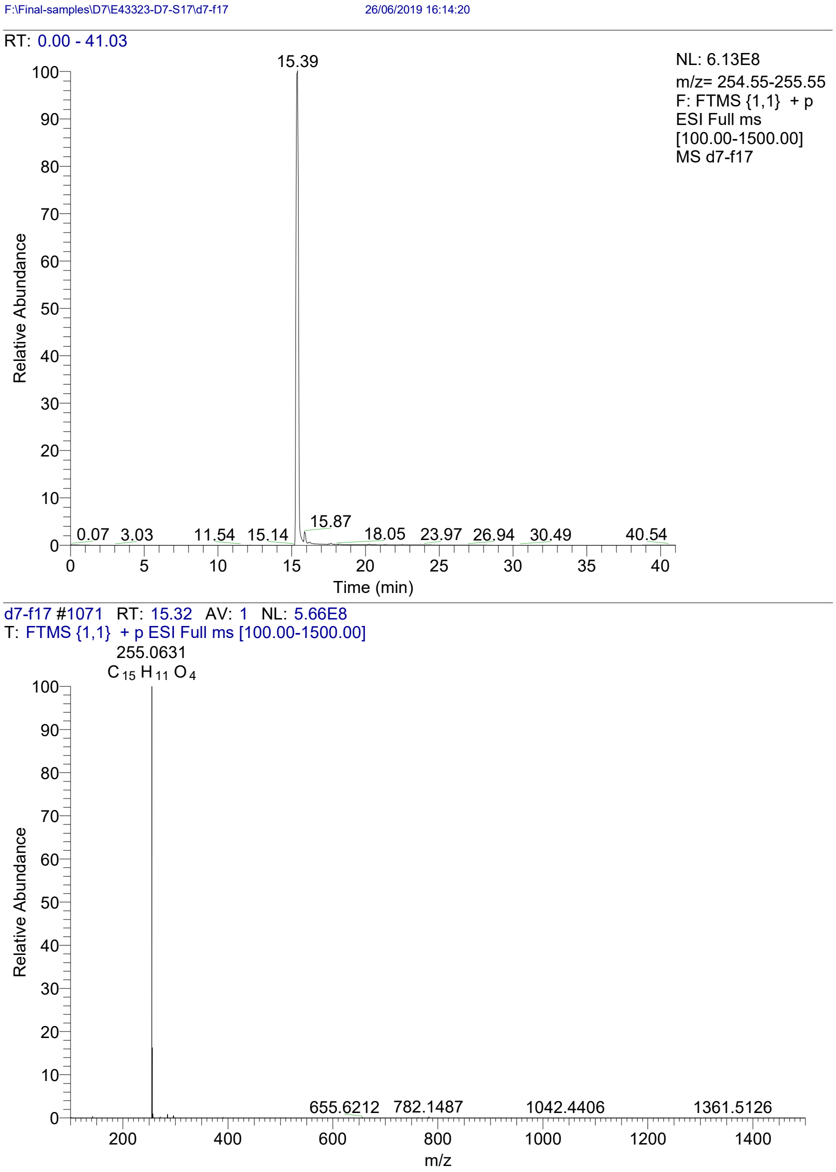

Figure S26. Extracted ion chromatogram and mass spectrum in positive ion mode for Chrysin.

Table S7 Chemical shifts for chrysin.

Position	**Chrysin**		Literature*	
	^{1}H δ ppm (mult, J in Hz)	^{13}C δ ppm, (mult)	^{1}H δ ppm, (mult, J in Hz)	^{13}C δ ppm, (mult)
1			-	-
2	-	163.64 (C)	-	163.6
3a	6.97(1H, s)	105.66 (CH)	6.94 (1H, s)	105.63
4	-	182.34 (C)		182.30
5	-	161.94 (C)	-	161.94
6	6.23 (1H, d)	99.5 (CH)	6.22(d)	99.49
7	-	164.91 (C)		164.91
8	6.53(1H, d)	94.60 (CH)	6.51(1H, d)	94.58
9	-	157.94 (C)	-	157.91
10	-	104.45 (C)	-	104.44
1`	-	131.2 (C)	-	131.19
2`	8.07 (1H, m)	126.88 (CH)	8.04(1H, m)	126.84
3`	7.58(1H, m)	129.60 (CH)	7.58(1H, m)	129.56
4`	7.55(1H, m)	132.48 (CH)	7.58(5H, m)	132.42
5`	7.58(1H, m)	129.60 (CH)	7.58(1H, m)	129.56
6`	8.07 (1H, m)	126.88 (CH)	8.04(1H, m)	126.84
7-OH	10.90 (1H, s)	-	10.90(s)	-

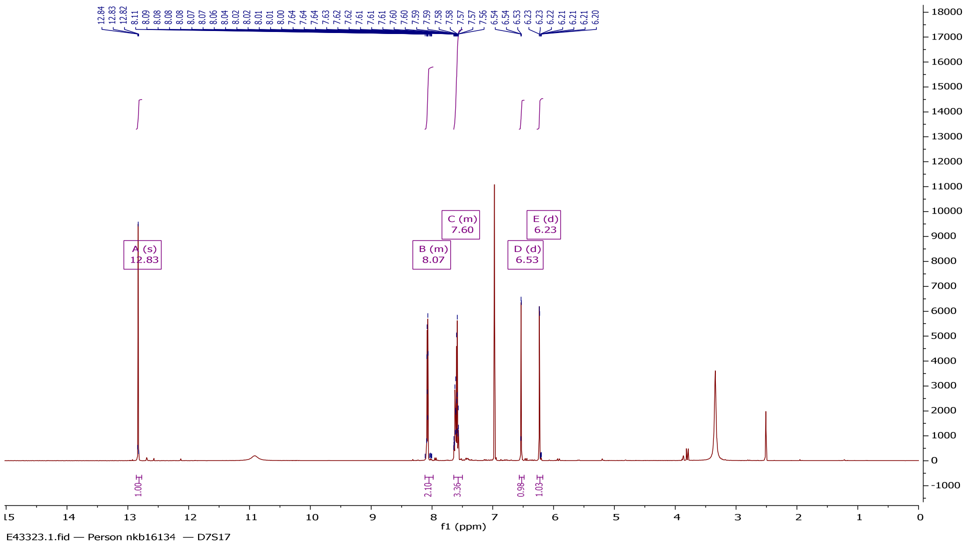

Figure S27. ^{1}H NMR (400 MHz) of Chrysin in DMSO d6.

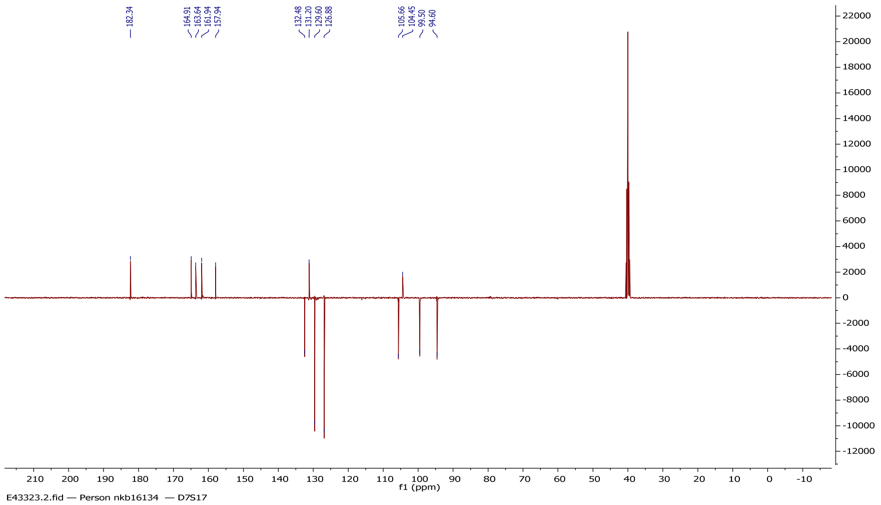

Figure S28. ^{13}C NMR (400 MHz) of Chrysin in DMSO d6.

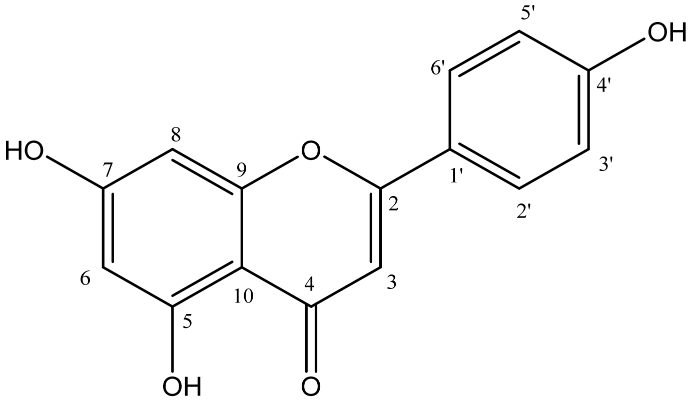

Figure S29. Chemical structure of Apigenin.

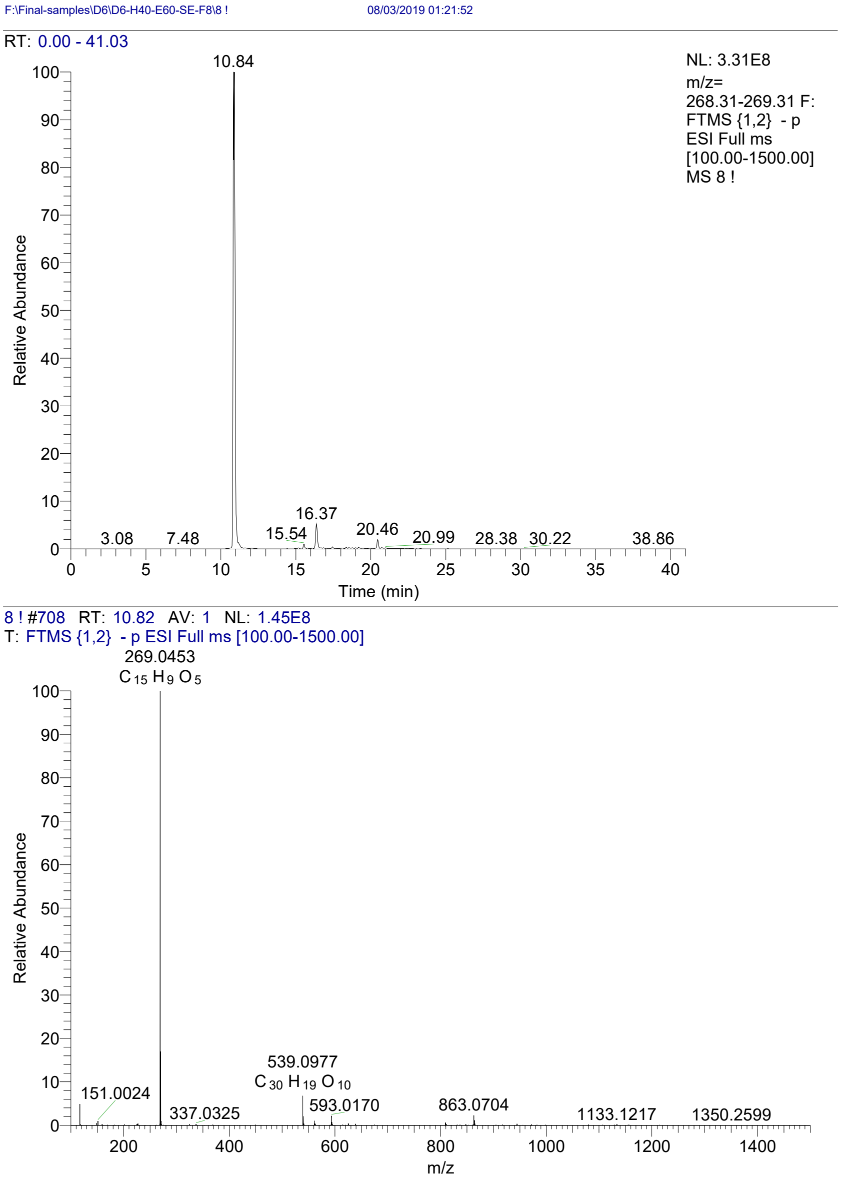

Figure S30. Extracted ion chromatogram and mass spectrum in negative ion mode for Apigenin.

Table S8. Chemical shifts for Apigenin.

Position	**Apigenin**		Literature*	
	^{1}H δ ppm (mult, J in Hz)	^{13}C δ ppm, (mult)	^{1}H δ ppm, (mult, *J* in Hz)	^{13}C δ ppm, (mult)
1			-	-
2	-	163.7 (C)	-	164.59
3	6.66 (1H, s)	103.27 (CH)	6.75 (1H, s)	103.31
4	-	182.20 (C)		182.19
5	-	161.51 (C)	-	161.62
6	6.23 (1H, d)	99.4 (CH)	6.22 (d)	99.30
7	-	164.9 (C)		164.19
8	6.53 (1H, d)	94.57 (CH)	6.51(1H, d)	94.42
9	-	157.88 (C)	-	157.77
10	-	104.3 (C)	-	104.18
1`	-	131.2 (C)	-	121.66
2`	7.97 (1H, m)	128.43 (CH)	7.90 (1H, d)	128.90
3`	7.05 (1H, m)	115.96 (CH)	6.92 (1H, d)	116.41
4`	-	161.7 (C)	-	161.93
5`	7.05 (1H, m)	115.96 (CH)	6.92 (1H, m)	116.41
6`	7.97 (1H, m)	126.9 (CH)	7.90 (1H, m)	128.90
7-OH	10.90 (1H, s)	-	10.75 (s)	-
5-OH	13.04 (s)	-	12.96 (s)	-
4-OH			10.40 (s)	

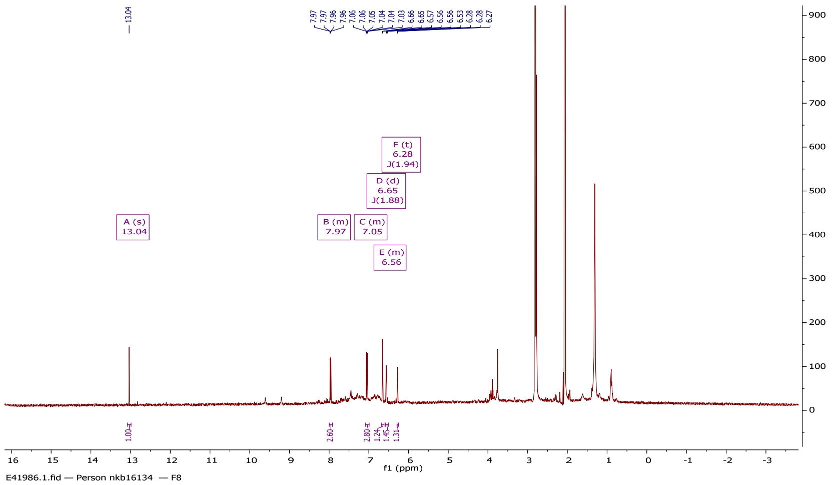

Figure S31. ^{1}H NMR (400 MHz) of Apigenin in Acetone d6.

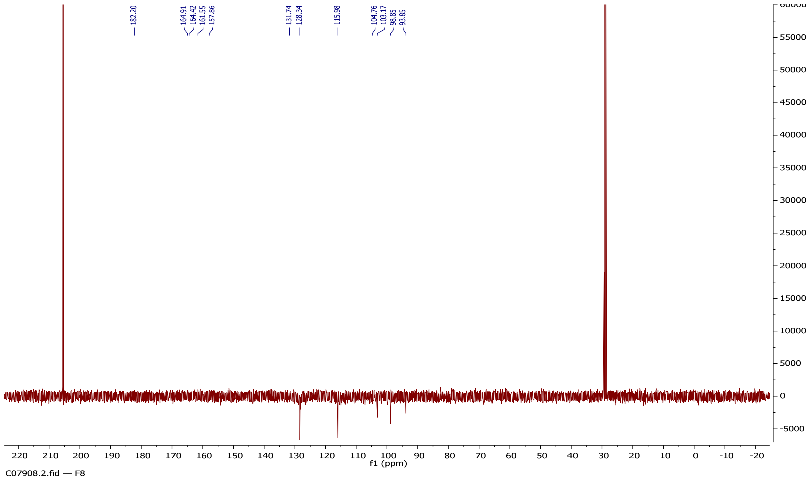

Figure S32. ^{13}C NMR (400 MHz) of Apigenin in Acetone d6.

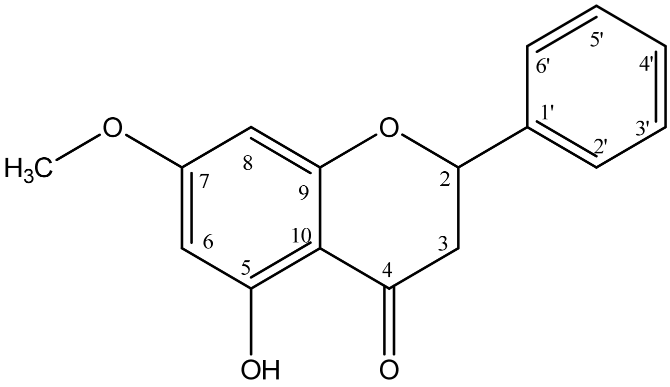

Figure S33. Chemical structure of Pinostrobin.

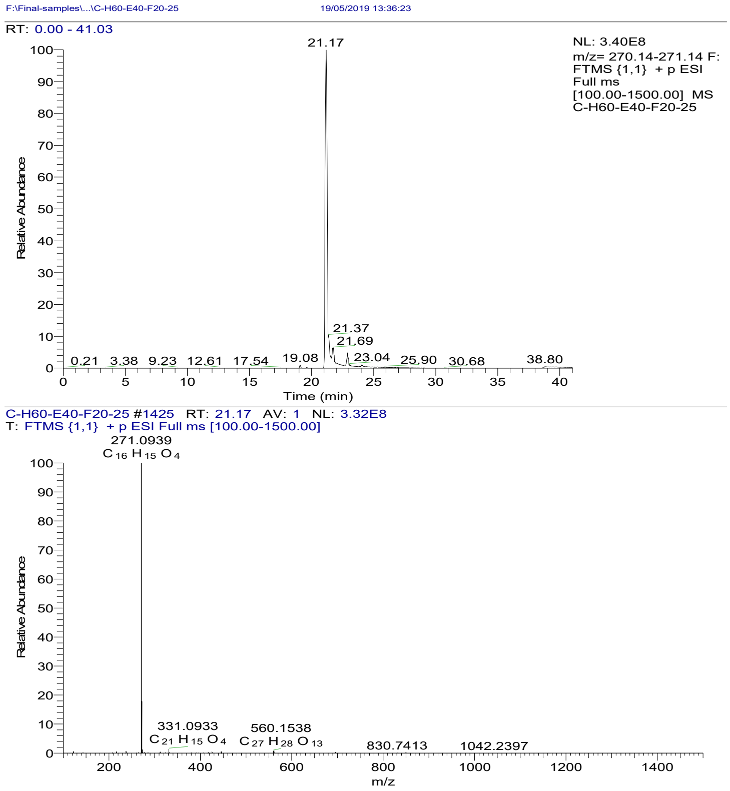

Figure S34. Extracted ion chromatogram and mass spectrum in positive ion mode for Pinostrobin.

Table S9. Chemical shifts for Pinostrobin

Position	**Pinostrobin**		Literature*	
	^{1}H δ ppm (mult, J in Hz)	^{13}C δ ppm, (mult)	^{1}H δ ppm, (mult, J in Hz)	^{13}C δ ppm, (mult)
1	-	-	-	-
2	5.43 (1H, dd, 13.0, 3.1)	79.4 (CH)	5.62(dd)	79.03
3a	2.83 (1H, dd, 17.2, 3.0)	43.8 (CH_2)	2.83 (dd)	42.63
3b	3.09 (1H, dd, 17.1, 3.1)		3.29(dd)	
4	-	196.5 (C)	-	196.92
5	-	161.4 (C)	-	163.71
6	6.08 (1H, d, 2.2)	95.4(CH)	6.00 (1H, d, *J* = 2.2)	95.24
7	-	168.3 (C)	-	167.94
8	6.07 (1H, d, 2.2)	94.4 (CH)	6.04 (1H, d, *J* = 2.2)	94.34
9	-	163.0 (C)	-	163.09
10	-	103.2(C)	-	103.10
1`	-	138.5 (C)	-	138.99
2`	7.45 (1H, m)	126.3 (CH)	-	127.06
3`	7.41 (1H, m)	129.0 (CH)	7.44 (5H, m)	129.06
4`	7.41 (1H, m)	129.0 (CH)		129.06
5`	7.41 (1H, m)	129.0 (CH)		129.06
6`	7.45 (1H, m)	126.3 (CH)		127.06
7-OCH_3	3.81 (3H, s)	55.8 (CH_3)	3.80(s)	56.33
5-OH	12.02 (s)	-	12.12 (1H, s)	-

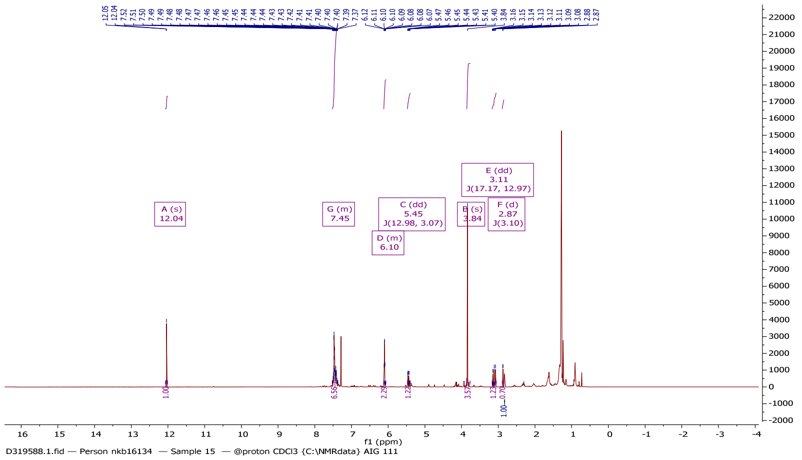

Figure S35. ^{1}H NMR (400 MHz) of Pinostrobin in $CDCl_3$.

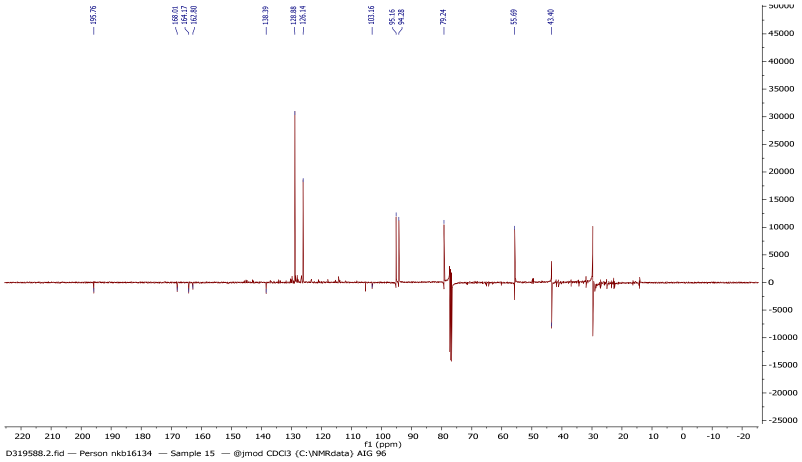

Figure S36. ^{13}C NMR (400 MHz) of Pinostrobin in $CDCl_3$.

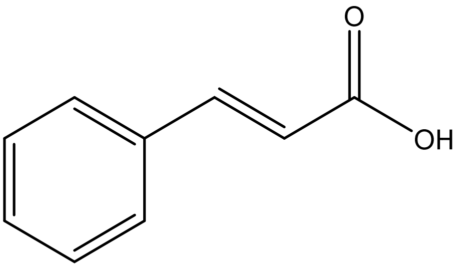

Figure S37. Chemical structure of Cinnamic acid.

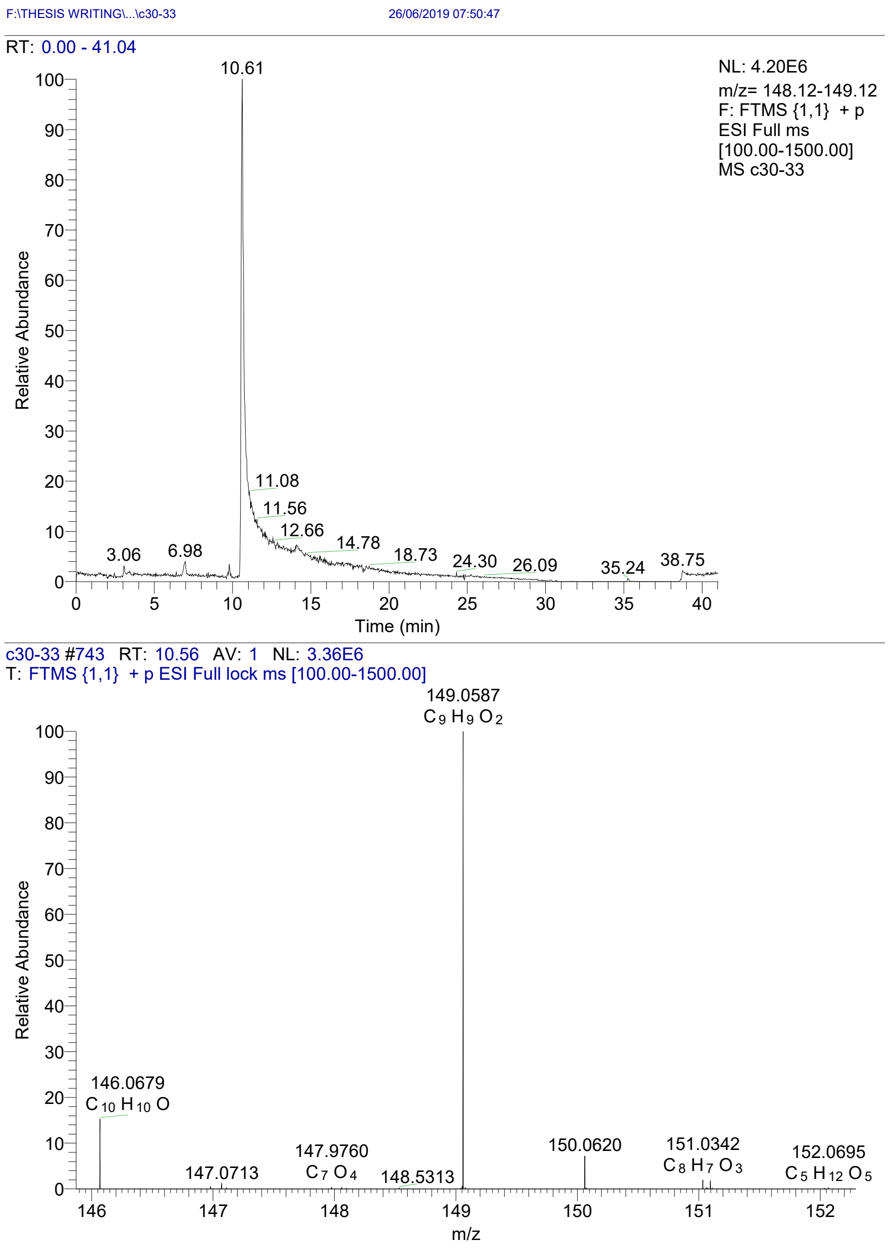

Figure S38. Extracted ion chromatogram and mass spectrum in positive ion mode for Cinnamic acid.

Table S10. Chemical shifts for Cinnamic acid.

Position	**Cinnamic acid**		Literature*	
	^{1}H δ ppm (mult, J in Hz)	^{13}C δ ppm, (mult)	^{1}H δ ppm, (mult, J in Hz)	^{13}C δ ppm, (mult)
1	-	171.8	-	168.00
2	6.46 (1H, d)	117.4 (CH)	6.53(d)	119.70
3	7.79 (1H, d)	147.2(CH)	7.59(d)	144.38
1′	-	134.2(C)	-	134.70
2′	7.58(1H, m)	128.4 (CH)	7.68(m)	128.65
3′	7.44 (1H, m)	129.0 (CH)	7.42(m)	129.36
4′	7.43(1H, m)	131.4(CH)	7.42(m)	130.67
5′	7.44 (1H, m)	129.0 (CH)	7.42(m)	129.36
6′	7.5(1H, m)	128.4 (CH)	7.68(m)	128.65
1-COOH	-	-	12.39(s)	-

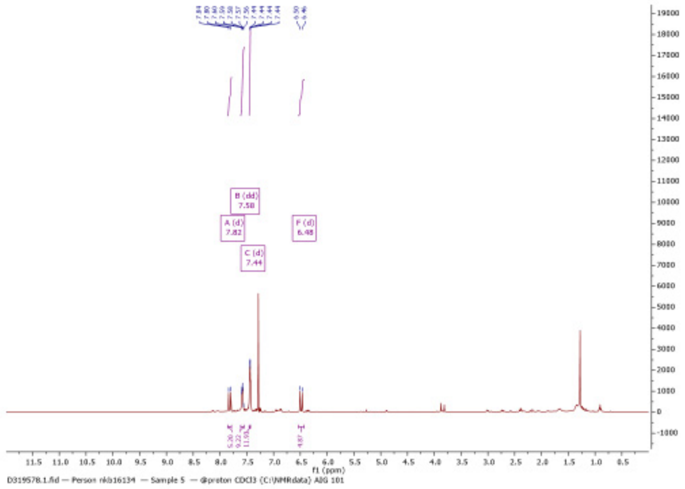

Figure S39. ^{1}H NMR (400 MHz) of Cinnamic acid in $CDCl_3$.

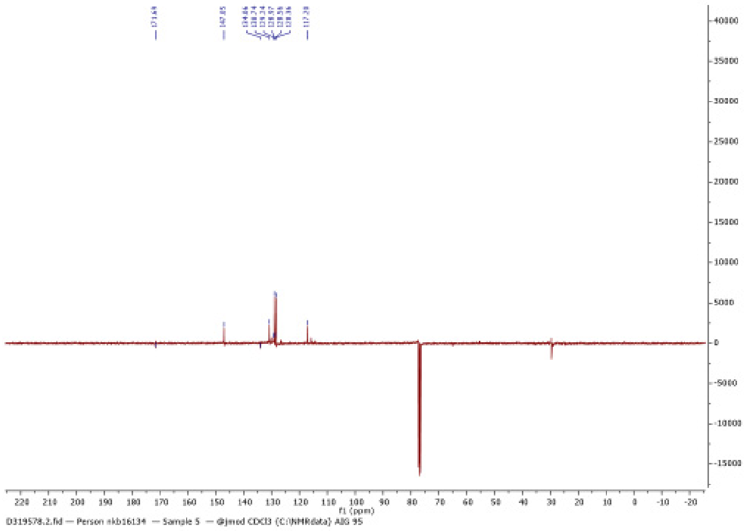

Figure S40. ^{13}C NMR (400 MHz) of Cinnamic acid in $CDCl_3$.

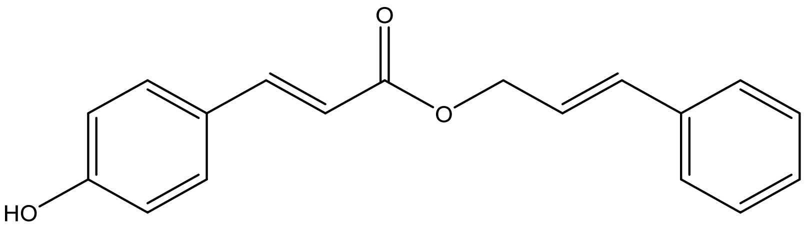

Figure S41. Chemical structure of Coumaric acid cinnamyl ester.

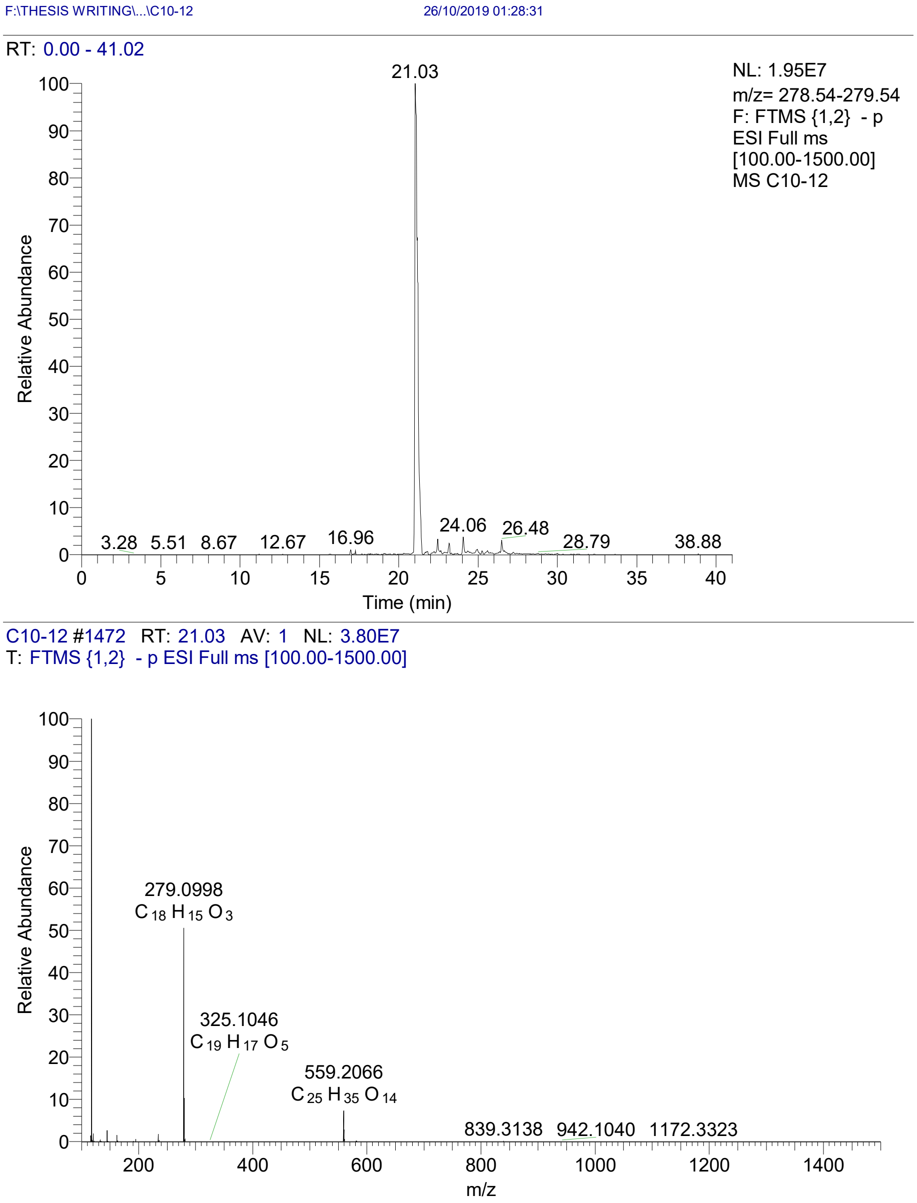

Figure S42. Extracted ion chromatogram and the mass spectrum in the negative ion mode for Coumaric acid cinnamyl ester.

Table S11. Chemical shifts for Coumaric acid cinnamyl ester.

Position	**Coumaric acid cinnamyl ester**		Literature*	
	^{1}H δ ppm (mult, J in Hz)	^{13}C δ ppm, (mult)	^{1}H δ ppm, (mult, *J* in Hz)	^{13}C δ ppm, (mult)
1	-	167.7 (C)	-	167.3
2	6.35 (1H, d)	114.9 (CH)	6.33 (1H, d, *J* = 15.8)	115.5
3	7.71 (1H, d)	145.4 (CH)	7.67(1H, d, *J* = 15.8)	144.9
4	-	126.86 (C)	-	128.1
5	7.44 (1H, 2.3)	130.1 (CH)	7.44 (1H, d,8.2)	130.1
6	6.88 (1H, d)	116.0 (CH)	6.85 (1H, d, *J* = 8.2)	116.0
7	-	158.24 (C)	-	157.8
8	6.88 (1H, d)	116.0 (CH)	6.85 (1H, d, *J* = 8.2)	116.0
9	7.44 (1H, 2.3)-	130.1 (CH)	7.44 (1H, d, *J* = 8.2)	130.1
1` a	4.91 (2H, d)	65.3 (CH_2)	4.86 (2H, d, *J* = 6.2)	65.1
1` b				
2`	6.37 (1H, d)	123.24 (C)	6.34 (1H, m)	123.5
3`	6.72 (1H, d)	134.38 (CH)	6.70 (1H, d, *J* = 15.8)	134.2
4`	-	136.2 (C)		136.4
5`	7.41 (1H, d)	126.66 (CH)	7.41 (1H, d, *J* = 7.6)	126.7
6`	7.36 (1H, d)	128.55 (CH)	7.34 (1H, d, *J* = 7.6)	128.7
7′	7.29 (1H, m,)	128.3 (CH)	7.25 (1H, m,)	128.7
8`	7.36 (1H, d)	128.55 (CH)	7.34(1H, d, *J* = 7.6)	128.7
9`	7.41 (1H, d)	126.66 (CH)	7.41(1H, d, *J* = 7.6)	126.7
7-OH	-	-	-	-

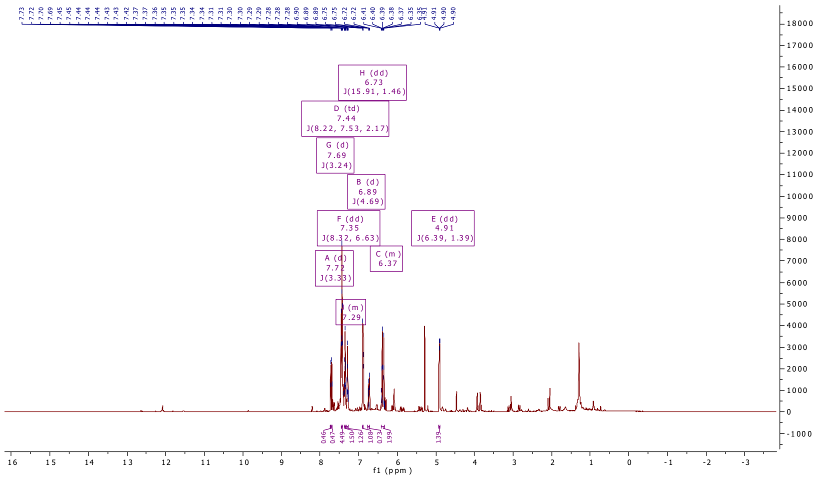

Figure S43. ^{1}H NMR (400 MHz) of Coumaric acid cinnamyl ester in $CDCl_3$.

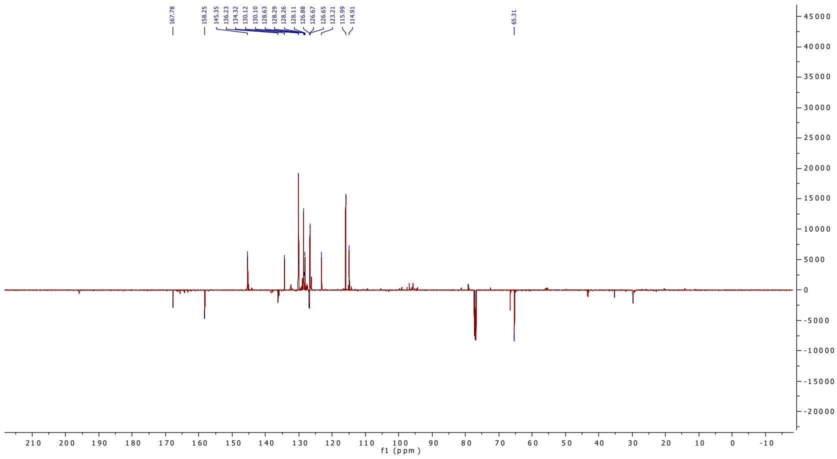

Figure S44. ^{13}C NMR (400 MHz) of Coumaric acid cinnamyl ester in $CDCl_3$.

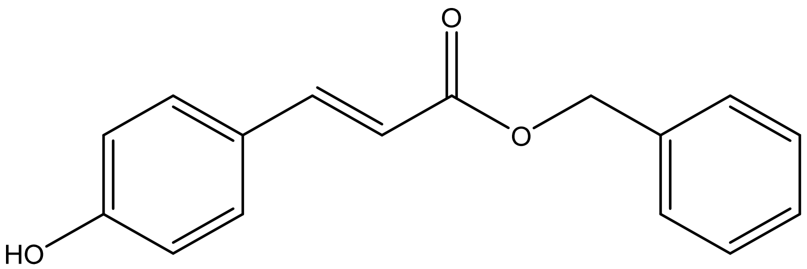

Figure S45. Chemical structure of Coumaric acid benzyl ester.

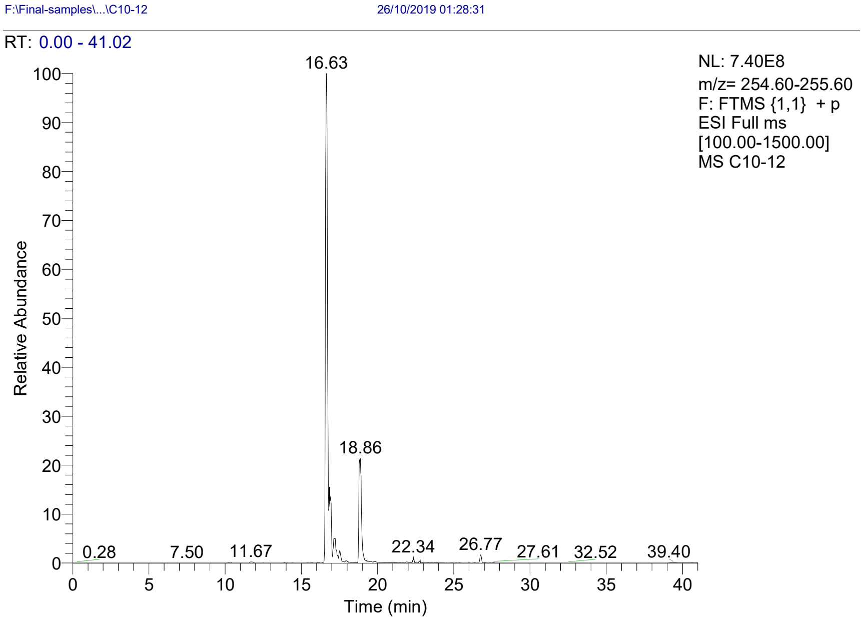

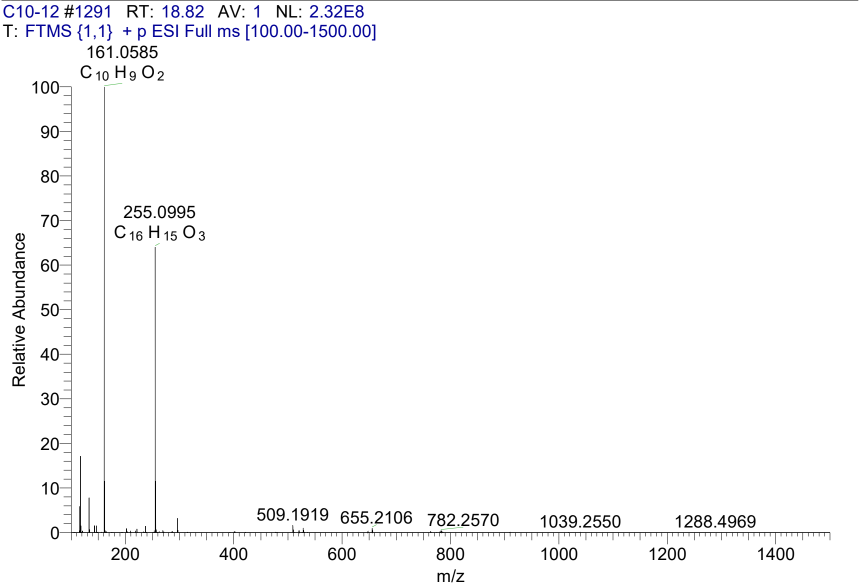

Figure S46. Extracted ion chromatogram and mass spectrum in positive ion mode for Benzyl p-coumarate.

Table S12. Chemical shifts for Coumaric acid benzyl ester

Position	**Coumaric acid benzyl ester**		Literature*	
	^{1}H δ ppm (mult, J in Hz)	^{13}C δ ppm, (mult)	^{1}H δ ppm, (mult, *J* in Hz)	^{13}C δ ppm, (mult)
1	-	167.7 (C)	-	167.4
2	6.35 (1H, d)	114.9 (CH)	6.34 (1H, d, *J* = 15.8 Hz)	115.2
3	7.70 (1H, d)	145.4 (CH)	7.67(1H, d, J= 15.8Hz)	145.0
4	-	126.9 (C)	-	127.1
5	7.44 (1H, d)	130.1 (CH)	7.42 (1H, d,8.2Hz)	130.0
6	6.88 (1H, d)	116.0 (CH)	6.83 (1H, d, *J* = 8.2Hz)	115.9
7	-	158.2 (C)	-	157.8
8	6.88 (1H, d)	116.0 (CH)	6.83 (1H, d, *J* = 8.2Hz)	115.9
9	7.44 (1H, d)	130.1 (CH)	7.42 (1H, d,8.2Hz)	130.0
1` a	5.29 (2H, s)	66.5 (CH_2)	5.25 (2H, d,6.2Hz)	66.1
1` b				
2`	-	136.0 (C)		136.1
3`	7.43 (1H, d)	126.7 (CH)	7.30 (1H, d, *J* =7.6Hz)	128.2
4`	7.36 (1H, d)	128.6 (CH)	7.34 (1H, d, *J* =7.6Hz)	128.7
5`	7.29 (1H, m,)	128.3 (CH)	7.28 (1H, m,)	128.7
6`	7.36 (1H, d)	128.55 (CH)	7.34 (1H, d, *J* =7.6Hz)	128.7
7′	7.43 (1H, d)	126.66 (CH)	7.40 (1H, d, *J* =7.6Hz)	128.6
7-OH	-	-	-	-

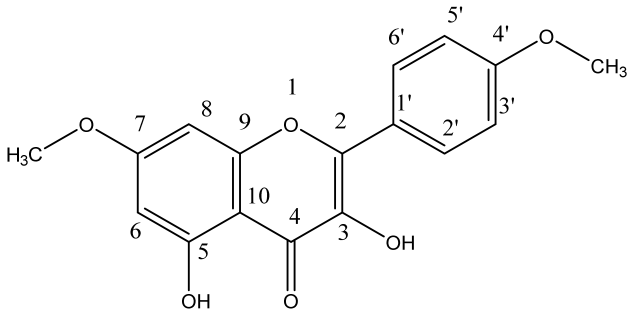

Figure S47. Chemical structure of 4′,7-dimethoxykaempferol.

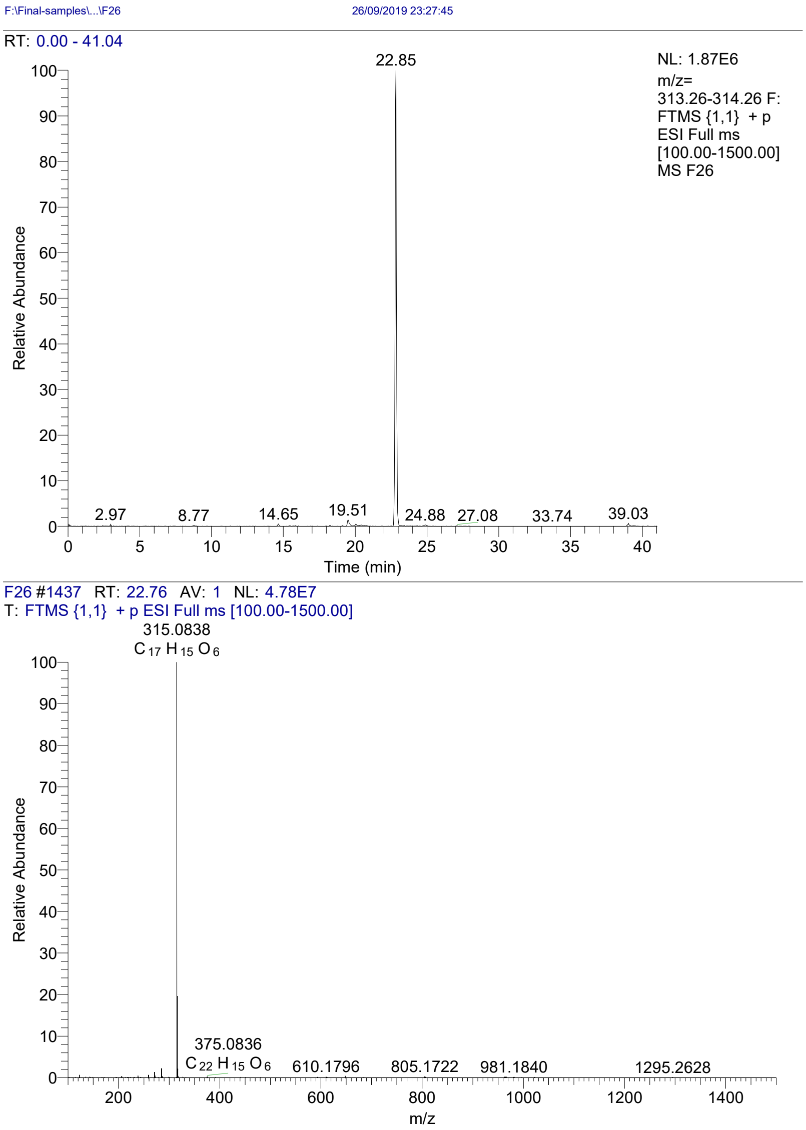

Figure S48. Extracted ion chromatogram and mass spectrum in positive ion mode for 4',7-Dimethoxykaempferol.

Table S13. Chemical shifts for 4',7-Dimethoxykaempferol.

Position	**4',7-Dimethoxykaempferol**		Literature*	
	^{1}H δ ppm (mult, J in Hz)	^{13}C δ ppm, (mult)	^{1}H δ ppm, (mult, J in Hz)	^{13}C δ ppm, (mult)
1	-	-	-	-
2	-	145.7 (C)	-	147.89
3	-	135.6 (C)	-	137.99
4	-	175.1(C)	-	177.25
5	-	160.9 (C)	-	162.23
6	6.40 (1H, d, 2.2)	98.0 (CH)	6.43 (IH, d, *J* = l.5 Hz)	98.05
7	-	165.7 (C)	-	165.45
8	6.52 (1H, d, 2.2)	92.1 (CH)	6.55 (l H, d, *J*=1.5 Hz)	92.62
9	-	156.8 (C)	-	157.72
10	-	103.9 (C)	-	105.58
1`	-	123.2 (C)	-	123.60
2`	8.20 (1H, d)	129.4 (CH)	8.25 (1H, d, *J* = 9.0Hz)	128.05
3`	7.06 (1H, d)	114.0 (CH)	7.08 (1H, d, *J* = 9.0 Hz)	114.5
4`	-	161.1 (C)		162.61
5`	7.06 (1H, d)	114.0 (CH)	7.08 (1H, d, *J* = 9.0 Hz)	114.5
6`	8.20 (1H, d)	129.4 (CH)	8.25 (1H, d, *J* = 9.0Hz)	128.05
7-OCH_3	3.91 (s)	55.9 (CH_3)	3.92 (s)	55.79
5-OH	11.76 (s)	-	12.75 (s)-	-
3-OH	6.61	-	-	-
4`-OCH_3	3.92 (s)	55.4 (CH_3)	3.92 (s)	55.52

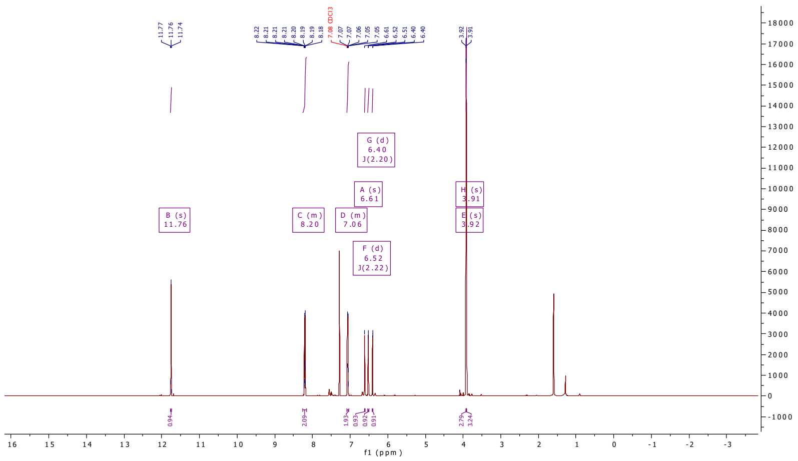

Figure S49. ^{1}H NMR (400 MHz) of 4',7-Dimethoxykaempferol in $CDCl_3$.

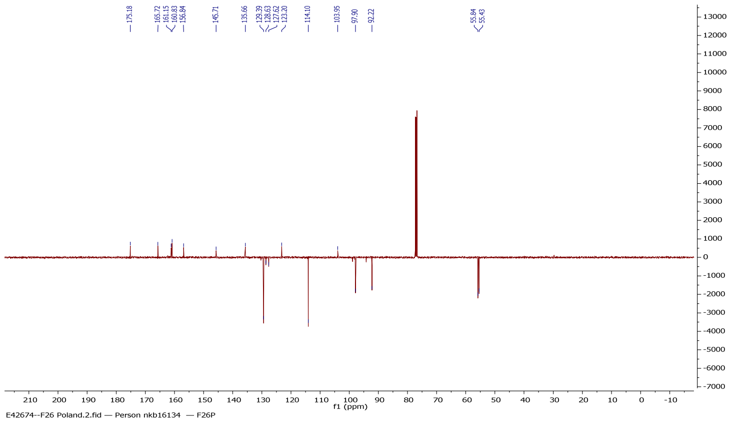

Figure S50. ^{13}C NMR (400 MHz) of 4',7-Dimethoxykaempferol in $CDCl_3$.

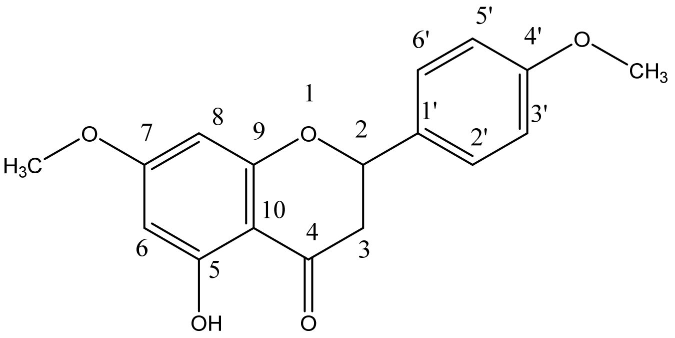

Figure S51. Chemical structure of Naringenin 4',7-dimethyl ether.

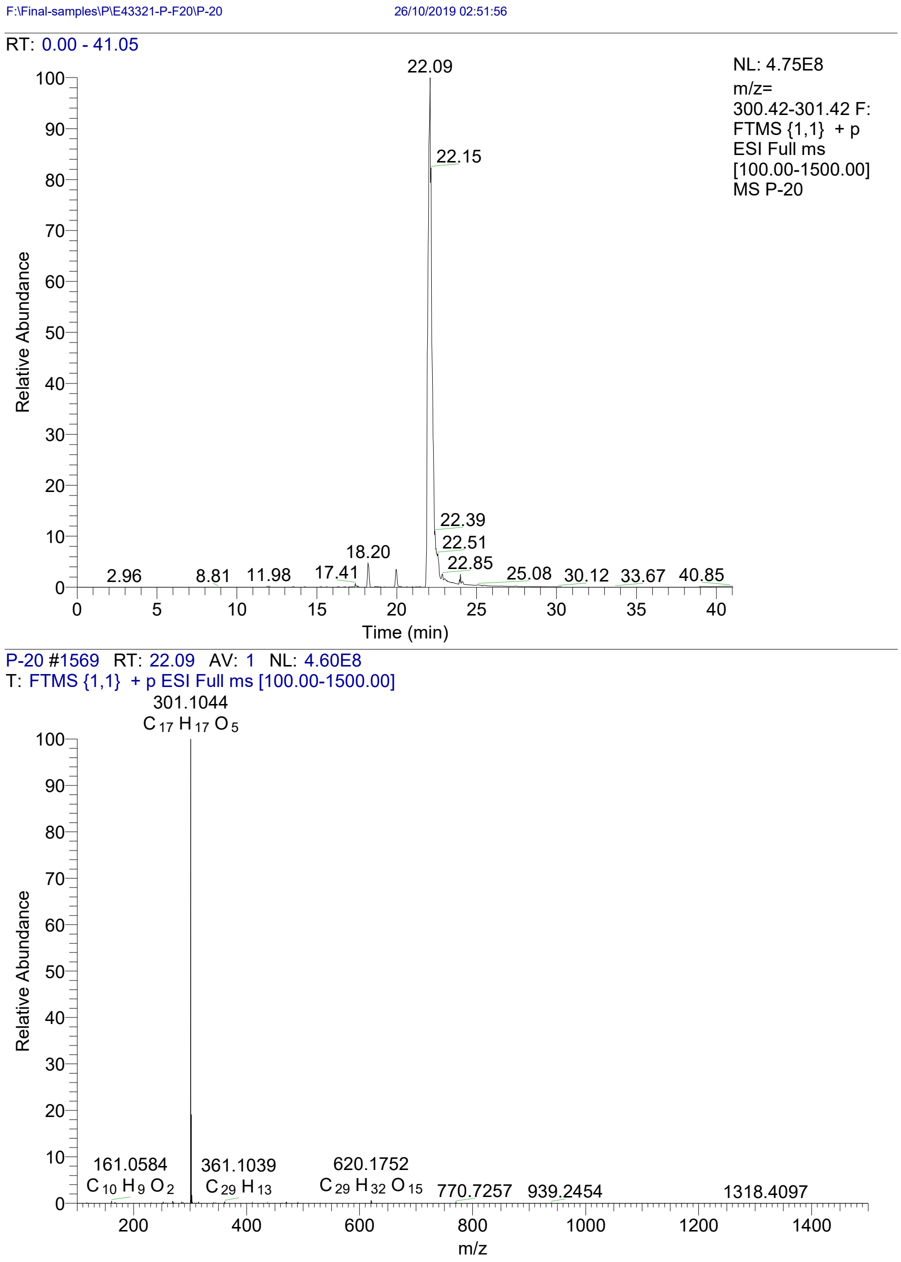

Figure S52. Extracted ion chromatogram and mass spectrum in positive ion mode for Naringenin 4',7-dimethyl ether.

Table S14. Chemical shifts for Naringenin 4',7-dimethyl ether.

Position	**Naringenin 4',7-dimethyl ether**		Literature*	
	^{1}H δ ppm (mult, J in Hz)	^{13}C δ ppm, (mult)	^{1}H δ ppm, (mult, J in Hz)	^{13}C δ ppm, (mult)
1	-	-	-	-
2	5.39 (1H, dd, 13.0, 2.7)	78.9 (CH)	5.37 (1H, d, *J* = 11.7)	79.7
3a	3.11 (1H, dd, 17.2, 13.0)	43.1 (CH_2)	3.12 (dd, J = 17.1, 13.1 Hz, 1H),	43.2
3b	2.81(1H, d, 17.2, 3.0)		2.79(17.2,3.0 Hz,1H)	
4	-	196.0 (C)	-	197.3
5	-	164.0 (C)	-	164.3
6	6.07 (1H, 2.3)	95.0 (CH)	6.04 (1H, d, *J* = 2.2)	95.3
7	-	167.0 (C)	-	168.7
8	6.10 (1H, 2.3)	94.2 (CH)	6.07 (1H, d, *J* = 2.2)	94.4
9	-	163.0 (C)	-	163.9
10	-	103.2 (C)	-	103.5
1`	-	130.4 (C)	-	131.6
2`	7.40 (1H, d)	127.7 (CH)	7.39(1H, d *J* = 8.6 Hz)	128.7
3`	6.98 (1H, d)	114.2 (C)	6.96(1H, d *J* = 8.7 Hz)	114.5
4`	-	160.1 (C)	-	160.8
5`	6.98 (1H, d)	114.2 (CH)	6.96(1H, d *J* = 8.7 Hz)	114.5
6`	7.40 (1H, m)	119.9 (CH)	7.39(1H, d *J* = 8.6 Hz)	128.7
7-OCH_3	3.83 (3H, s)	55.7 (CH_3)	3.80 (s, 3H)	56.0
4'-OCH_3	3.86 (3H, s)	55.4 (CH_3)	3,89 (s, 3H)	55.4
5-OH	12.04 (s)	-	-	

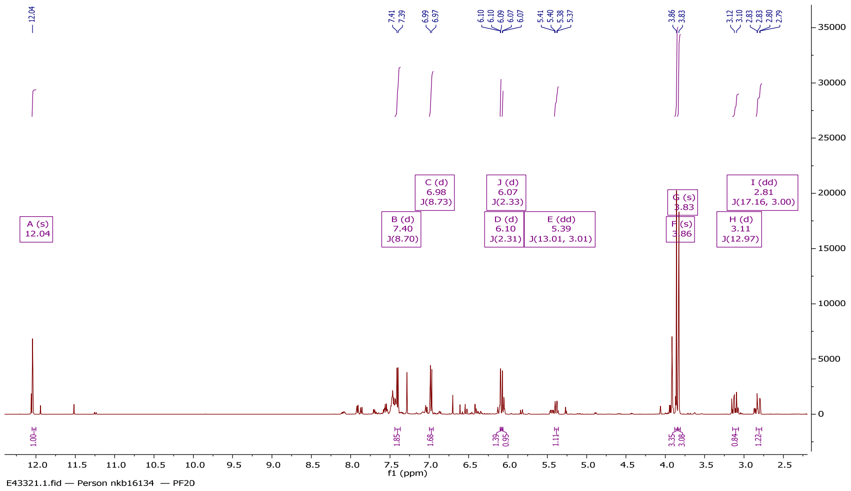

Figure S53. ^{1}H NMR (400 MHz) of Naringenin 4',7-dimethyl ether in $CDCl_3$.

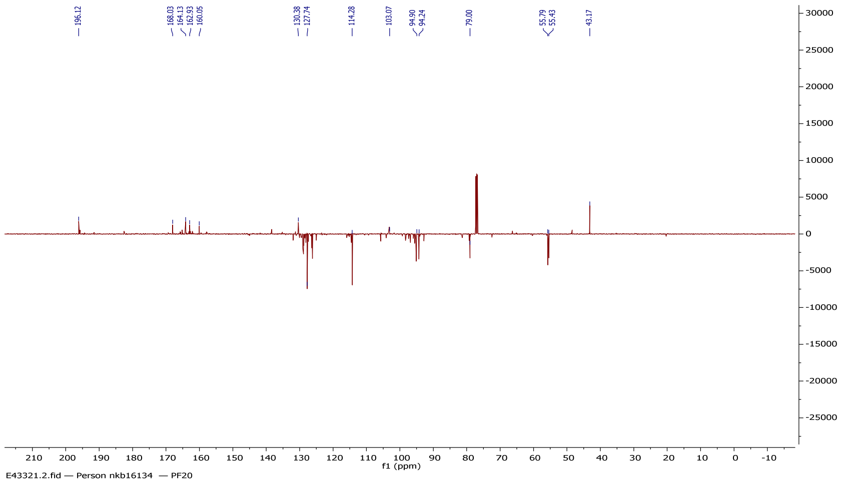

Figure S54. ^{13}C NMR (400 MHz) of Naringenin 4',7-dimethyl ether in $CDCl_3$.
